# Harnessing the potential of probiotics in the treatment of alcoholic liver disorders

**DOI:** 10.3389/fphar.2023.1212742

**Published:** 2023-06-09

**Authors:** Garima Mishra, Pradeep Singh, Mulugeta Molla, Yohannes Shumet Yimer, Subas Chandra Dinda, Phool Chandra, Bhuvnesh Kumar Singh, Samuel Berihun Dagnew, Abraham Nigussie Assefa, Amien Ewunetie

**Affiliations:** ^1^ Pharmaceutical Chemistry Unit, Department of Pharmacy, College of Health Sciences, Debre Tabor University, Debre Tabor, Ethiopia; ^2^ Pharmacology and Toxicology Unit, Department of Pharmacy, College of Health Sciences, Debre Tabor University, Debre Tabor, Ethiopia; ^3^ Social Pharmacy Unit, Department of Pharmacy, College of Health Sciences, Debre Tabor University, Debre Tabor, Ethiopia; ^4^ School of Pharmacy, The Neotia University, Kolkata, India; ^5^ Department of Pharmacology, Teerthanker Mahaveer College of Pharmacy, Teerthanker Mahaveer University, Moradabad, India; ^6^ Faculty of Pharmacy, MET Group of Institutions, Moradabad, India; ^7^ Clinical Pharmacy Unit, Department of Pharmacy, College of Health Sciences, Debre Tabor University, Debre Tabor, Ethiopia

**Keywords:** probiotics, gut-liver axis, microbiome, alcoholic liver disease, dysbiosis

## Abstract

In the current scenario, prolonged consumption of alcohol across the globe is upsurging an appreciable number of patients with the risk of alcohol-associated liver diseases. According to the recent report, the gut-liver axis is crucial in the progression of alcohol-induced liver diseases, including steatosis, steatohepatitis, fibrosis, cirrhosis, and hepatocellular carcinoma. Despite several factors associated with alcoholic liver diseases, the complexity of the gut microflora and its great interaction with the liver have become a fascinating area for researchers due to the high exposure of the liver to free radicals, bacterial endotoxins, lipopolysaccharides, inflammatory markers, etc. Undoubtedly, alcohol-induced gut microbiota imbalance stimulates dysbiosis, disrupts the intestinal barrier function, and trigger immune as well as inflammatory responses which further aggravate hepatic injury. Since currently available drugs to mitigate liver disorders have significant side effects, hence, probiotics have been widely researched to alleviate alcohol-associated liver diseases and to improve liver health. A broad range of probiotic bacteria like *Lactobacillus*, *Bifidobacteria*, *Escherichia coli*, *Sacchromyces*, and *Lactococcus* are used to reduce or halt the progression of alcohol-associated liver diseases. Several underlying mechanisms, including alteration of the gut microbiome, modulation of intestinal barrier function and immune response, reduction in the level of endotoxins, and bacterial translocation, have been implicated through which probiotics can effectively suppress the occurrence of alcohol-induced liver disorders. This review addresses the therapeutic applications of probiotics in the treatment of alcohol-associated liver diseases. Novel insights into the mechanisms by which probiotics prevent alcohol-associated liver diseases have also been elaborated.

## 1 Introduction

The liver has an inherent role in the body, particularly in metabolism and detoxification. Meanwhile, it is vulnerable to many drugs, chemicals, environmental pollutants, and infections (Gu and Manautou, 2012). Alcohol drinking has now become a global trend, generating health-related problems among people. In addition, drinking alcohol has a negative impact on social and economic status as well. Although, regular intake of excessive alcohol may have detrimental effects on nearly all body organs, the liver gets the highest degree of tissue damage owing to its prime role in ethanol metabolism (Osna et al., 2017). Moreover, alcohol is linked to a variety of diseases, including cardiovascular (hypertension, arrhythmia, heart attack) (Rehm et al., 2010; Larsson et al., 2020), neurological (dementia, Huntington’s disease, multiple sclerosis, depression, epilepsy) (Pervin and Stephen, 2021), and various types of cancer (liver, oropharynx, esophagus, colon, rectum, breast) (Shield et al., 2013).

It has been stated that Europeans frequently develop liver cirrhosis due to uncontrolled alcohol consumption. Overconsumption of alcohol or heavy drinking can also be referred to as alcohol use disorder (AUD), which represents the major cause of alcoholic liver disease (ALD). Notably, ALD are the most common cause of death due to high alcohol intake (Shield et al., 2013). As per the World Health Organization (WHO), alcohol abuse and alcohol addiction cause approximately 3.8% of deaths and 4.6% of disability-adjusted life-years across the world (Siddiqi et al., 2020). Heavy drinking, as described by the National Institute of Alcohol Abuse and Alcoholism and the Centers for Disease Control and Prevention (CDC), is ingestion of 8 or more drinks per week for females and 15 or more drinks per week for males (Siddiqi et al., 2020). A review of the most recent WHO global data, alcoholic hepatitis (AH) and liver cirrhosis have a rather high fatality rate, with rates exceeding 50% in severe acute AH (World Health Organization, 2018). It has been stated that approximately 60% of liver cirrhosis occurs as a result of alcohol abuse in Europe and North America. Moreover, nearly 80,000 people die as a result of alcohol-induced hepatocellular carcinoma (HCC). Therefore, liver transplantations are common occurrences in Europe and North America (Addolorato et al., 2020). Cirrhosis-related mortality rates are anticipated to triple by 2030, owing largely to a surge in the prevalence of ALD and nonalcoholic fatty liver disease (Tapper and Parikh, 2018). Heavy drinkers are more likely to develop a wide range of liver ailments, the most frequent of which are steatosis, hepatitis, and cirrhosis. Hepatic steatosis, characterized by fat deposition in hepatocytes, is the earliest reaction to heavy drinking (Peng et al., 2017).

**FIGURE 1:**
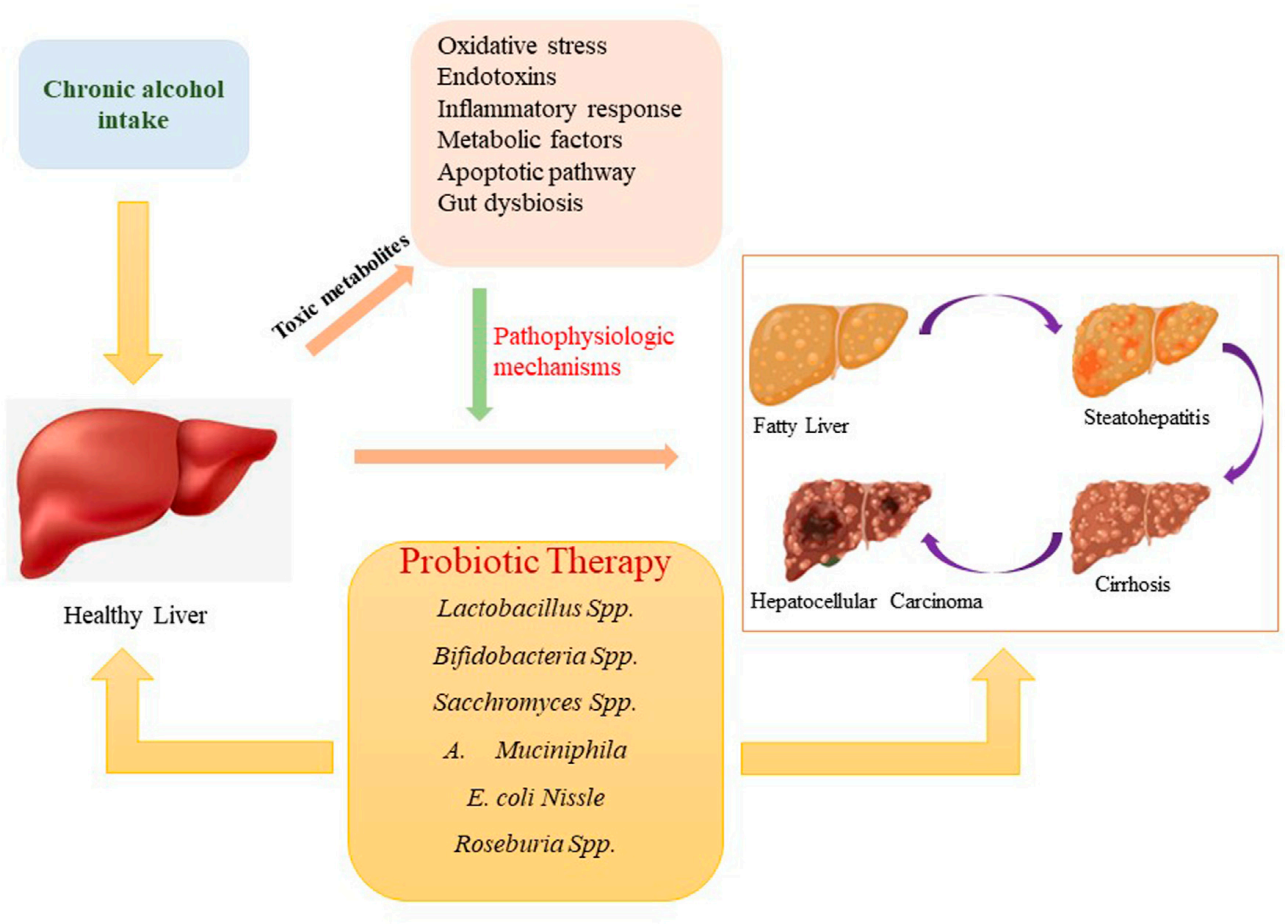
Progression of alcohol-associated liver disorders and probiotics effect on liver health.

To date, the pathogenesis of alcoholic hepatic damage is unexplained at both the cellular and molecular level. Furthermore, there are insufficient effective treatments or FDA-approved medications accessible to treat alcoholic liver disorders. Alcohol withdrawal (also known as abstinence) can, however, fix moderate hepatic lesions caused by alcohol but not chronic stages such as cirrhosis. In addition to abstinence, some convectional treatment approaches, such as corticosteroids and nutritional support, have been proposed to cure ALD related complications (Gao and Bataller, 2011; Jackson, 2021). Probiotics have been deeply explored in recent years as functional or novel agents to improve hepatic functioning hepatic function and cure ALD. The favourable effects of several probiotic strains on alcoholic liver disorders, as well as possible underlying mechanisms (Figure 1), are summarized in this paper.

## 2 Alcoholic liver disease

Heavy alcohol intake and sedentary lifestyles are key contributors to the widespread chronic condition known as ALD in both developed and developing countries. Hepatic lesions and dysfunctions produced by excessive alcohol consumption are the most serious outcomes. ALD constitutes a series of liver-related toxicity, including steatosis (i.e., fatty liver), alcoholic steatohepatitis (ASH), liver fibrosis, cirrhosis, and HCC (Ohashi et al., 2018; Sharma and Arora, 2020; Subramaniyan et al., 2021). Hepatic steatosis is the intrahepatic accumulation of lipid content, mainly triglycerols (TAG), in the liver. The amount of fat within the hepatocytes is used to assess liver steatosis: grade 0 (healthy, 5%), grade 1 (mild, 5%–33%), grade 2 (moderate, 34%–66%), and grade 3 (severe, >66%) (Nassir et al., 2015). Approximately 90% of those who consume alcohol exhibit signs of steatosis (Ishak et al., 1991; Ohashi et al., 2018). It is distinguished by the presence of microscopically discernible lipid droplets within the hepatocytes. This condition can be quickly treated by adopting a healthy lifestyle that includes physical activity and nutritional changes. (Nassir et al., 2015). ASH, the second stage of steatosis ALD, arises as a result of prolonged use of alcohol. It affects nearly 10%–35% of alcohol drinkers. The predominant histopathological hallmarks of ASH include polymorphonuclear cell infiltration and hepatic necrosis (Frazier et al., 2011). Furthermore, alcohol consumption may precipitate the progression of more severe stages such as liver fibrosis and cirrhosis, increasing the risk of consequences such as variceal hemorrhage, hepatic encephalopathy, and renal failure. Cirrhosis is the last stage of liver fibrosis, which involves the replacement of diseased tissues with a collagenous scar in hepatic stellate cells (HSC). Cirrhosis is predicted to affect 8%–20% of chronic alcoholic drinkers (Purohit and Brenner, 2006; Farooq and Bataller, 2016). The main implications of liver cirrhosis are regenerative nodular hepatic echotexture, surrounded by fibrotic bands, distortion of the hepatic vasculature, and loss of hepatic functions (Zhou et al., 2014). Alcoholic cirrhosis is a significant risk factor for the advancement of HCC (Tarao et al., 2019). According to a recent estimate, around 1%–2.0% of HCC cases with alcoholic cirrhosis are diagnosed each year (Stickel, 2015; Stickel et al., 2017). Aside from alcohol consumption, other risk factors such as gender inequity, genetic polymorphism, race and ethnicity, the hepatitis virus, diabetes, and obesity can all contribute to cirrhosis and HCC (Fattovich et al., 2004). The subsequent subsection explains the underlying mechanisms of ALD.

## 3 Alcohol metabolism

**FIGURE 2:**
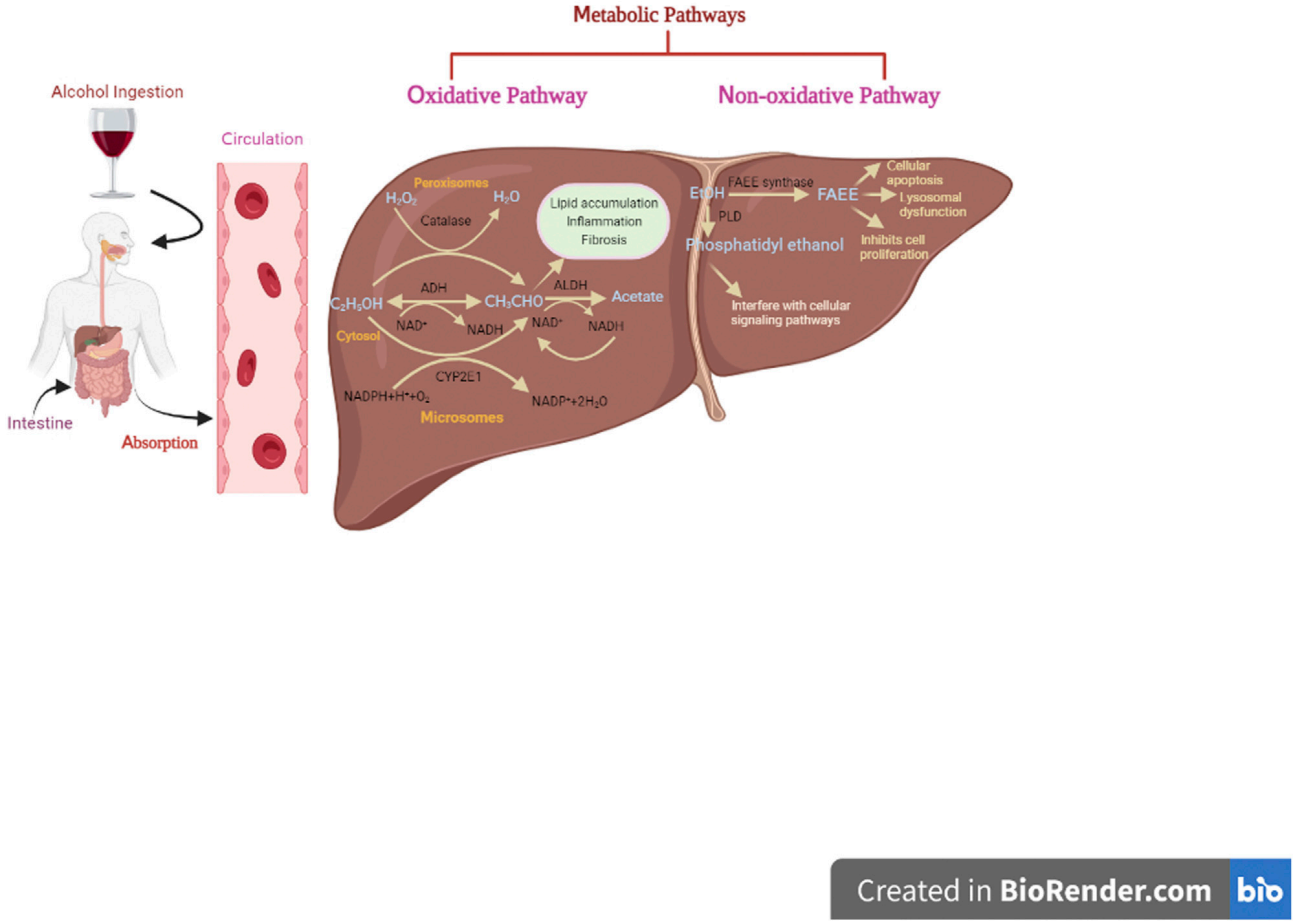
Formation of toxic metabolites from ethanol through oxidative and non-oxidative pathways.

Blood alcohol concentration (BAC), a key parameter, is used to determine the effect of alcohol on several tissues, which further dependent on alcohol absorption, distribution, metabolism, and excretion (Zakhari, 2006). Once alcohol is ingested in the body, 90% of it gets absorbed by the small intestine and delivered to the liver through the portal vein, while the remaining 10% is eliminated through sweat, breath, and urine (Holford, 1987; Cederbaum, 2012). However, there are certain factors like sex, age, race, diet, and physical exercise, and medication that affect the rate of alcohol elimination from the body (Cederbaum, 2012). The liver plays a vital role in alcohol metabolism due to its high abundance of metabolizing enzymes (Cederbaum, 2012). Alcohol is metabolized in the liver by both oxidative and non-oxidative mechanisms as highlighted in Figure 2.

### 3.1 Oxidative pathway

The oxidative pathway is regarded as a major route for alcohol metabolism, accounting for more than 90% of alcohol elimination (Pawan, 1972; Mackus et al., 2020). There are three metabolic routes for oxidative metabolism of alcohol, each involving the presence of some essential enzymes such as alcohol dehydrogenase (ADH), acetaldehyde dehydrogenase (ALDH), and CAT. ADH, present in hepatocytes cytoplasm, catalyzes the conversion of alcohol into acetaldehyde using nicotinamide adenine dinucleotide (NAD+) as a co-factor, while ALDH further oxidizes acetaldehyde into acetate. This is the most relevant metabolic pathway at lower concentrations of alcohol in blood and tissue fluids.

The second pathway involves the microsomal cytochrome P450 2E1 (CYP2E1) enzyme, particularly when alcohol consumption is high (more than 10 mol/L). Acetaldehyde is the major metabolite mediated through CYP2E1. Additionally, other highly reactive oxygen species (ROS) and hydroxyethyl species are also produced that may contribute to oxidative stress. These radicals generated through CYP2E1 cause detrimental effects on proteins, lipids, nucleic acids, and other biomolecules.

The peroxisomal catalase (CAT) is the third oxidative metabolic system for ethanol metabolism. This oxidative route is considered as a minor pathway in alcohol metabolism since it requires hydrogen peroxide (Zakhari, 2006; Heier et al., 2016; Mackus et al., 2020; Hyun et al., 2021).

### 3.2 Nonoxidative pathway

The remaining 10% of alcohol is metabolized *via* non-oxidative pathways in other tissues, including brain, pancreas, and heart, where oxidative pathways do not exist (Laposata and Lange, 1986; Manautou et al., 1992; Mackus et al., 2020). However, the resultant metabolites (ethyl sulfate, ethyl nitrite, ethyl phosphate, and FAEEs, etc.) of this pathway may have some pathological and diagnostic significance (J. Dinis-Oliveira, 2016). Formation of ethyl glucuronide (EtG) and ethyl sulfate (EtS) involves phase II metabolic reactions, i.e., glucuronidation and sulfate conjugation, respectively. Glucuronidation is catalyzed by uridine diphosphate (UDP)-glucuronosyltransferases (UGTs) (Rowland et al., 2013), while sulfate conjugation takes place through sulfotransferases (Kurogi et al., 2012), both are phase II enzymes. Both of these metabolites are physiologically inactive since their excretion is aided by the phase II reaction (Rowland et al., 2013). Furthermore, enzymatic esterification of alcohol with fatty acids results in the formation of fatty acid ethyl esters (FAEEs). Two enzymes, namely, FAEE synthase and acyl-CoA-ethanol-O-acyltransferase (AEAT), participate in the synthesis of FAEEs (Grigor and Bell, 1973; Treloar et al., 1996). FAEEs are potentially dangerous metabolites that cause cellular apoptosis, lysosome instability, suppression of cell proliferation, and mitochondrial malfunction, and are useful indicators for alcohol consumption (Rowland et al., 2013). Phosphatidylethanols (PEth), on the other hand, are generated *via* transphosphatidylation of phospholipids with ethanol in the presence of phospholipase D (PLD). Under normal conditions, PLD hydrolyzes membrane phospholipids to produce phosphatidic acid (PA). PA plays a critical role in a variety of cellular processes, including endocytosis, membrane trafficking, cytoskeletal reorganization, proliferation, and migration. Nonetheless, PEth formation has a negative impact on some enzyme activities, cellular signaling pathways, and bio-membranes (Heier et al., 2016).

### 3.3 Molecular mechanisms of alcohol-associated liver diseases

ALD pathogenesis has been established by a number of underlying molecular pathways. These mechanisms include the involvement of alcohol and its metabolites in oxidative stress induction as well as the role of inflammatory responses. (Kong et al., 2019). Furthermore, some papers have focused on the impact of genetic determinants, microRNA, and some other risk factors implicated in ALD pathogenesis. (Namachivayam and Valsala Gopalakrishnan, 2021).

#### 3.3.1 Role of metabolites in hepatic damage

Acetaldehyde, the most common oxidative alcohol-derived metabolite, plays a wide range of roles in liver injury, including lipid deposition, inflammation, fibrosis, and carcinogenesis. (Hyun et al., 2021). Acetaldehyde forms adducts with a variety of proteins, DNA, and other biomolecules, promoting lipid peroxidation, GSH depletion, and mitochondrial toxicity (Farfán Labonne et al., 2009; Setshedi et al., 2010). Furthermore, this toxic compound contributes to hepatocyte apoptosis, the activation of innate and adaptive immunity, neutrophil infiltration, and the inhibition of liver regeneration (Gao and Bataller, 2011). Figure 2 depicts the role of acetaldehyde in ALD. Acetate, a breakdown product of acetaldehyde, on the other hand, is quickly released from the liver into circulation and bio-transformed into carbon dioxide *via* Kreb’s cycle. However, other studies report that acetate may elevate the levels of proinflammatory cytokines in macrophages and stimulate inflammation in AH patients (Shen et al., 2009; Kendrick et al., 2010).

#### 3.3.2 Role of oxidative stress

Excessive ROS generation during the course of ethanol metabolism, as mediated by the dehydrogenase system and microsomal system, often induces oxidative stress, which subsequently leads to hepatic damage and eventually, ALD (Galicia-Moreno and Gutiérrez-Reyes, 2014).

In addition, ROS serve as key components for the production of harmful compounds such as malondialdehyde (MDA) and 4-hydroxy-2-nonenal (HNE) by lipid peroxidation. Both MDA and HNE also form adducts with proteins (Cederbaum et al., 2009). Apart from these adducts, acetaldehyde, when combined with MDA, interacts with proteins to generate the malondialdehyde-acetaldehyde-protein adduct. All of these adducts are associated with inflammatory processes, immune responses, and the advancement of liver disorders (Willis et al., 2002).

The microsomal system, on the other hand (active CYP2E1), stimulates the generation of ROS, notably superoxide and hydroxyl radicals, resulting in oxidative stress and cell death. Oxygen radicals can cause hepatocyte injury by releasing tumor necrosis factor (TNF) and lipopolysaccharides (LPS) (Cichoz-Lach and Michalak, 2014). Moreover, the mitochondrial and peroxisomal enzymes, (e.g., acyl-CoA dehydrogenase, carnitine palmitoyl transferase-1), that are primarily responsible for β-oxidation, are peroxidized by ROS formed by CYP2E1. Alterations in these enzymes promote fatty acid buildup, which leads to the first stage of ALD, hepatic steatosis (Cichoz-Lach and Michalak, 2014).

Some studies have demonstrated that oxidative stress has a detrimental effect on liver mitochondria. ROS may alter the permeability of the mitochondrial membrane and transition potential, resulting into release of proapoptotic factors (*e.g.,* cytochrome C and caspase-3) and reduced production of ATP. Irreversible changes in mitochondria induced by ROS have also been related to impaired protein synthesis due to ribosomal injury. ROS may also disrupt microsomal and lysosomal membranes, increasing lipid peroxidation and lowering glutathione sulfhydryl and glutathione-S-transferase activity. As a result, oxidative stress invariably leads to cell death. Following steatosis, hepatic fibrosis and cirrhosis develop as a result of stellate cell rebuilding and extracellular matrix activation mediated by ROS. Alterations in stellate cells can be induced by the activation of matrix metalloproteinases. Furthermore, oxidative stress adds to the loss of regeneration potential of mature hepatocytes, which results in hepatic progenitors (Ambade and Mandrekar, 2012; Tan et al., 2020).

#### 3.3.3 Role of endotoxins

It has been documented that overconsumption of alcohol accelerates the multiplication of Gram-negative bacteria in the colon, resulting in an imbalance of intestinal flora and buildup. Furthermore, acetaldehyde accumulation from alcohol metabolism increases tyrosine phosphorylation in tight and adherent junctions. These events increase intestinal permeability, allowing translocation of endotoxins to the liver. Accumulation of endotoxins triggers inflammatory alterations in the hepatic and other tissues (Kavanaugh et al., 2005; Purohit et al., 2008). Another study has shown that alcohol and its metabolites induce several nuclear transcription factors, like nuclear factor-kappa B (NF-kB) and inducible nitric oxide synthase (iNOS), also increase intestinal permeability when binding to tubulin and activating intracellular non-specific protease C. This causes microtubule damage, thereby disrupting the intestinal barrier functions (Groebner and Tuma, 2015; Kong et al., 2019; Nowak and Relja, 2020). Similarly, bacterial endotoxins, through toll-like receptors (TLR), activate the Kupffer cells and macrophages in the liver, contributing to the production of cytokines and other inflammatory mediators and compromising immune regulatory functions. It promotes endotoxin leakage into the bloodstream in ALD patients (Szabo, 2015). Blood endotoxins activate inflammatory and Kupffer cells, which hinder phagocytosis and stimulate proliferation of HSC by the massive release of cytokines (IL-1, IL-17, TNF-α, osteopontin), and free radicals (Gao and Bataller, 2011).

#### 3.3.4 Role of hepcidin regulation

According to the study, another hallmark of ALD is iron accumulation or overload in the liver, which is mediated by a number of regulatory mechanisms. The alcohol-induced downregulation of hepcidin production in the liver is the most common underlying mechanism implicated in ALD and is attributed to iron deposition (Ioannou et al., 2004; Harrison-Findik, 2007). Hepcidin is a key regulator for iron homeostasis (Nemeth et al., 2004). Furthermore, iron and alcohol contribute to oxidative stress and lipid peroxidation by generating free radicals and releasing pro-inflammatory cytokines (Tsukamoto et al., 1995; Pietrangelo, 1998). Oxidative stress stimulates transferrin receptor 1 (TfR1) levels that further enhance intestinal iron absorption. Collectively, both increased iron absorption and deposition exacerbate liver damage (Harrison-Findik, 2007; Silva et al., 2017).

#### 3.3.5 Role of adipose tissue

It has been reported that adipose tissue is a key regulator for almost all metabolic pathways. Adipose tissue is largely involved in glucose metabolism and maintains glucose homeostasis. Recent research suggests that chronic alcohol intake may influence adipose tissue metabolic processes such as enhanced lipolysis, an imbalance in the insulin-glucose system, and hypersecretion of adipokines like resistin and lipocalin 2, which results in the production of inflammatory cytokines (Baraona and Lieber, 1979; Pravdova and Fickova, 2006). Among a wide range of adipokines, adiponectin, leptin, and resistin have been reported to be associated with ALD. Adiponectin plays an important role in glucose metabolism, fatty acid oxidation, and insulin sensitization *via* modifying the AMP-activated protein kinase (AMPK) pathway. Several experimental studies have recorded a decrease in adiponectin level in chronic ALD (Shen et al., 2010; Tian et al., 2014). Leptin perform a vital role in food intake, utilization of energy, lipolysis, and fatty acid oxidation. Research has shown that chronic intake of alcohol elevates the level of leptin protein and its receptor in adipose tissue (Obradovic and Meadows, 2002; Stern et al., 2016; Steiner and Lang, 2017) As a result, people with alcoholism have higher liver fat levels but lower overall fat mass, which influences leptin circulating levels (Martínez-Uña et al., 2020). Resistin is also expressed in liver cells, and its production appears to rise as liver damage progresses (Da Silva et al., 2018). It has been documented that resistin level is increased in alcoholic patients due to inflammation (Kema et al., 2015). Interestingly, increased resistin level in serum is also associated with obesity and type 2 diabetes mellitus (Pravdová et al., 2007). Persistent alcohol intake has a significant impact on lipid and glucose homeostasis. Altogether, adipokines and impaired lipid metabolism cause an inflammatory response by releasing pro-inflammatory mediators such as IL-6, TNF-α, MCP-1 from adipose tissue, resulting in liver injury.

#### 3.3.6 Role of apoptosis

Long-term consumption of alcohol or alcohol abuse provokes massive ROS generation, resulting in hepatic apoptosis through oxidative stress and inflammatory conditions (Ishii et al., 2003; Chakraborty et al., 2012). In addition, alcohol abuse also contributes to mitochondrial dysfunction, endoplasmic reticulum stress, decreased methylation, and altered proteasomal functions. The aforementioned factors stimulate the apoptosis of hepatocytes. Mitochondrial-dependent apoptosis can be triggered by ROS by inhibiting the phosphorylation of α-serine/threonine-protein kinase (AKT). This downregulates the level of cyclinD1 *via* the inactivating glycogen synthase kinase 3 beta (GSK3-β)/Wnt/β-catenin signaling pathway in hepatic cells and thus causes cell arrest. Furthermore, activation of other signaling molecules such as nuclear factor kappa-light-chain-enhancer of activated B (NF-kB), apoptosis signal-regulating kinase 1 (ASK1), and c-Jun N-terminal kinases (JNK)/P38 may lead to mitochondria-dependent apoptosis (Kong et al., 2019). Furthermore, caspase-8 and caspase-9 pathways have also been implicated in ethanol-induced hepatocyte apoptosis. Both of these pathways downregulate the level of caspases 3 and 7, which eventually enforce apoptotic cascades (Lalaoui and Vaux, 2018). Nonetheless, a more recent study has demonstrated the role of an anti-apoptotic protein/caspase-binding protein, namely, X-linked inhibitor of apoptosis (XIAP) which directly binds caspases 3,7 and 9 and inactivates them (He et al., 2021). Therefore, XIAP could be an effective therapeutic intervention for halting alcohol-associated liver diseases.

#### 3.3.7 Gut microbiota

The human gastrointestinal tract (GIT), one of the largest interfaces, consists of a complex and wide range of microorganisms that have marked influence on nutrition and human health. Nearly, 100 trillion microorganisms predominantly bacteria but also fungi, protozoa, and viruses have been recorded to be present in the human GIT (Liang et al., 2018; Valdes et al., 2018). Bacteria from three major groups, Firmicutes, Bacteroidetes, and Actinobacteria, account for the majority of the microorganisms in the gut microbiota (Liang et al., 2018). Despite the negative impact of pathogenic microorganisms on human health, the microbiome serves as a key player in the treatment of numerous human diseases like obesity, diabetes, cardiovascular diseases, cancer, IBS, neurological disorders, and many more (Ding et al., 2019; Chen J. et al., 2021). In recent decades, researchers have developed keen interest in the gut microbiome owing to its multifarious functions, including its role in metabolism, boosting immunity and CNS functions, and colonization resistance (host protection against colonization by pathogenic invaders). Nevertheless, observational findings have illustrated that certain factors, such as dietary components, stress, consumption of drugs and alcohols, host factors, may disrupt the normal functioning of gut microbiota, affecting the host’s health and wellness. This phenomenon is known as dysbiosis (Knight and Nigam, 2019). Moreover, dysbiosis increases the number of pathogenic microorganisms (pathobionts), resulting in production of toxic metabolites or products. These microbial derived metabolites might have a detrimental effect on the host, leading to diverse range of illnesses, including hepatic diseases. Recent data pointed out that dysfunction of the intestinal barrier, fatty acid metabolism, immunity, translocation of toxic elements produced by pathogenic bacteria, bile acid homeostasis, AhR (Aryl hydrocarbon receptor) signaling, and FXR (farnesoid X receptor) signaling are some of the key players leading to ALD development *via* intestinal dysbiosis (Chen et al., 2022). In such cases, microbiome-based therapies have the potential to improve metabolic health and management of metabolic diseases.

## 4 Probiotics

The emergence of probiotics as a novel complementary therapy for a multiple range of chronic diseases has received escalating attention over the past few decades in healthcare, research, and the public domain. The term “probiotic” is derived from a Greek word meaning “for life”. Ferdinand Vergin, in 1954, first discovered the term probiotic and also narrated the beneficial effects of useful microorganisms against the harmful effects of antibiotics. Probiotics can be defined in a variety of ways, as can be seen in Table 1 (Schrezenmeir and De Vrese, 2001; Parvez et al., 2006; Lee et al., 2008; Markowiak and Ślizewska, 2017).

**TABLE 1:** Definitions of probiotics by various authors.

Scientist name	Year	Definition
Lilly and Stillwell	1965	Probiotics are microorganisms that promote the growth of other microorganisms
Guarner and Schaafsma	1998	Stated the critical benefits of probiotics in sufficient doses exerting their beneficial effects
Sperti	1971	Probiotics are tissue extracts that induce microbial growth
Parker	-	Probiotics are the substances that maintain gut microbiome balance
Fuller	1989	Probiotics are viable microorganisms that have potential benefits for their host
Havenaar	1992	Probiotics are live, single, or blend microbial cultures that, when administered to an animal/human, offer health benefits to the host by ameliorating the properties of indigenous microbiota
Salminen	-	A probiotic is a viable microbial culture/cultured dairy product that positively affects the nutrition and health of the host
Schaafsma	-	Probiotics are living microbes that ingested orally in an appropriate amount, have health benefits beyond their inherent basic nutrition
Gibson and Robertfroid	1995	Probiotics are regarded as non-digested food components that maintain the host’s health by enhancing the growth or activity of either single or multiple types of microorganisms present in the GIT.

The concept of probiotics was then accepted by the FAO (the United Nations Food and Agriculture Organization) and the WHO (the World Health Organization) in 2002. As per FAO and WHO, probiotics are living microorganisms that, when supplied in an adequate amount to the host, confer health benefits (Ontario, 2006; Jiang et al., 2021). The International Scientific Association for Probiotics and Prebiotics (ISAPP, 2014) has endorsed this definition of probiotics, which is frequently used in scientific papers (Hill et al., 2014).

These candidates have a promising impact on public health by altering the composition of the gut microbiota and thus ameliorating the quality of life, particularly in the elderly population.

Previous data have demonstrated the vital role of probiotics in a variety of chronic conditions such as diabetes, cancer, hypertension, inflammatory disorders, immune disorders, respiratory diseases, GIT disorders, liver disorders, allergy, and a variety of infections (Iqbal et al., 2014; Nazir et al., 2018; Manzoor et al., 2022).

In this context, innovative non-invasive therapeutic approaches such as probiotics are being researched for the treatment of numerous diseases and preserving the health of human beings (Gebrayel et al., 2022). Interestingly, some probiotics have currently been reported as mitigation strategy against various bacterial and viral infections, including COVID-19 diseases (Silva et al., 2020; Yang et al., 2020; Harper et al., 2021; Kurian et al., 2021; Manzoor et al., 2022). Moreover, probiotics have been reported to show their potential role in numerous GIT disorders like inflammatory bowel disease (IBD), gastroenteritis, diarrhea, colitis, celiac disease, and many more (Stavropoulou and Bezirtzoglou, 2020). Subsequent insights sheds new light on probiotics’ potential therapeutic role in ALD (Liu C. et al., 2021).

### 4.1 Salient features of probiotics

The advent of probiotics in the scientific arena offers tremendous potential for treating several diseases, including ALD. In this context, probiotics have been engineered to restore useful gut or intestinal microbiota, which contributes to the development of strong gut immune system, the production of short-chain fatty acids and vitamins, the digestion of dietary content, and the inhibition of the colonization of pathogenic microorganisms (Hemarajata and Versalovic, 2013; Markowiak-Kopeć and Śliżewska, 2020; Pham et al., 2021). This section provides the ideal characteristics and features of probiotics for exerting their potent therapeutic effects. It is extremely important that the probiotic strain stays alive in the environment where it is thought to be active. Probiotics must be able to endure the stomach and duodenum environments. Besides, it should be able to boost the immune system, allowing for better intestinal function. Probiotics should not be affected by pancreatic juice, bile, or hydrochloric acid. In order to achieve better health outcomes, they must also be stable during fabrication and storage. The strain should be able to grow and colonize at this specific area for maximal activity. Moreover, probiotics should not be pathogenic, allergenic, or mutagenic (Nagpal et al., 2012; Harzallah and Belhadj, 2013).

### 4.2 Mechanism of probiotics in alcohol-associated liver diseases

Insights into the mechanistic approaches of probiotic effects against ALD still remain undefined. Although, some important underlying mechanisms through which probiotics exert their action have been illustrated, including through antioxidant activity, alteration in hepatic lipid metabolism, downregulation of inflammatory mediators, improvement of the intestinal epithelial barrier function, modulation of the mucosal immune system, regulation of gut microbiota.

**FIGURE 3:**
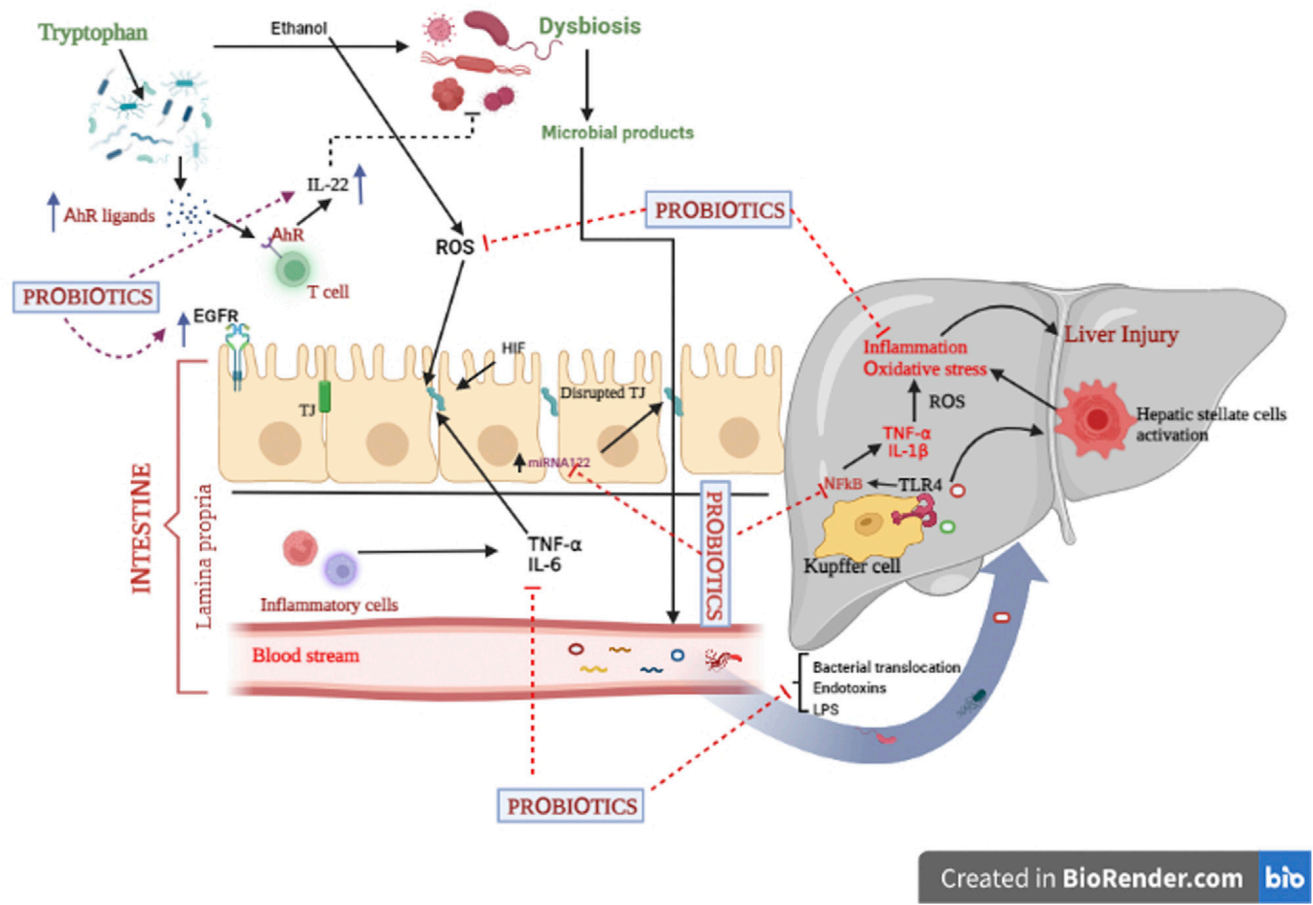
Various underlying mechanisms of probiotics implicated in alcoholic liver diseases.

Of the aforesaid mechanistic approaches of probiotics, the proceeding segment only describes the most common mechanisms, i.e., maintenance of intestinal epithelial barrier function and regulation of gut microbiota by probiotics (Figure 3) (Mazziotta et al., 2023; Zhang et al., 2023).

#### 4.2.1 Improvement of the intestinal epithelial barrier function

In fact, intestinal epithelial barrier dysfunction can be a major hallmark for the development of alcohol-associated liver diseases. Reportedly, several factors like pathogenic microorganisms, excessive generation of free radical spp., and production of inflammatory markers are crucial elements for disruption of the intestinal barrier function (Madsen, 2012; Rose et al., 2021). It has been reported that gut commensal bacteria substantially contribute to maintaining the intestinal barrier’s integrity. Intestinal probiotics like *Lactobacillus*, *Bifidobacteria*, *Escherichia coli.* Strains, and *A. muciniphila* successfully improve gut homeostasis and promote health. Accumulating data revealed that surface layer proteins (SLPs), capsular polysaccharides (CPS), lipopolysaccharides, lipoteichoic acid, pili, and flagella, recognized as surface components, particularly interact with specific pattern recognition receptors (PRRs) such as NOD- and toll-like receptors (NLRs and TLRs). This binding further modulates many signaling pathways like NF-kB, MAPK, and PPAR-gamma in the intestinal epithelial cells. Additionally, cellular protease-dependent signalling cascades also result in the production of chemokines (CCL2) and cytokines (IL-4, IL-10, IL-11, and IL-13), which reduce inflammation and enhance intestinal barrier integrity (Madsen, 2012; Liu et al., 2020). On the other hand, enterocyte barrier junction is characterized by the presence of specific junctions called tight junctions. These junctions comprise two important transmembrane proteins, namely, occludin and claudins. Upregulated expression of these proteins improves the barrier’s integrity and thus alleviates hepatic injury. Micro RNA 122a (miR122a) is another target of the tight junction protein occludin (Yan et al., 2013). Occludin is decreased due to the alcohol-induced upregulation of miR122a. Probiotic treatment normalize occludin levels by lowering miR122a expression in the ALD model (Zhao et al., 2015). Surprisingly, intestinal epithelial cells exert immunomodulatory function in the gut through their association with immune cells (Vinderola et al., 2005; Ohland and MacNaughton, 2010; Madsen, 2012). Another key player that contributes to intestinal barrier integrity is hypoxia-inducible factor (HIF). This transcription factor mainly stimulates the expression of the intestinal trefoil factor (ITF), xenobiotic clearance by P-glycoprotein, and some other nucleotide cascades (Colgan and Taylor, 2010). Furthermore, upregulated HIF-1α could be another target to prevent the alcohol-induced gut leakiness and the translocation of bacteria and their toxic components to the liver (Shao et al., 2018). Another piece of evidence has pointed out that probiotics, *via* epidermal growth factor receptor (EGFR)-dependent mechanism, maintain gut permeability and integrity against alcohol-induced liver injury (Shukla et al., 2018). Transactivation of EGFR can significantly prevent apoptotic events and restore the barrier function in intestinal epithelial cell (IEC) (Yan et al., 2013). Taken together, probiotics might be claimed as a novel strategy for ALD *via* improved intestinal barrier functions.

#### 4.2 Regulation of gut microbiota

Recent investigations have shown that an imbalance in the gut microbiome not only affects the GIT but also disturbs the liver’s functions through the gut-liver axis, causing various disorders including ALD. Hence, alterations of gut microbiota have become a significant target for exploring the underlying mechanisms of probiotics. Many bacterial spp. including *Lactic acid bacteria* (LAB), *Bifidobacteria*, and *A. muciniphila* have been experimentally investigated to preserve the gut microflora and prevent alcohol-induced liver diseases (Gu et al., 2019; Liu et al., 2021). Notably, alcohol consumption stimulates gut dysbiosis and overgrowth of pathogenic microorganisms and lowers AhR formation (Bajaj et al., 2014; Gabbard et al., 2014). Research has shown that AhR ligands (metabolites of tryptophan) modulate the gut microbiota through IL-22 production by intestinal T cells (Agus et al., 2018; Natividad et al., 2018; Hendrikx et al., 2019). Moreover, gut microbiota imbalance promotes intestinal permeability, a decrease in TJ protein status, and immune related dysfunctions, which further cause hepatic inflammation. Probiotic supplementation reduces the growth of pathogenic Gram-negative bacteria, improves phagocytic activity, stimulates IgA production, and thus boosts cellular immunity (Gu et al., 2019). Probiotics, through regulation of gut flora bacteria, suppress the levels of hepatic endotoxins, which result in decreased production of pro-inflammatory markers (IL-6, TNF-α, IFNγ etc.) *via* downregulated expression of NF-kB (O’Sullivan, 2008). Conclusively, probiotics exert their beneficial effects against ALD by stimulating the balance of gut commensals.

## 5 Common probiotics for alcohol-associated liver diseases

Probiotics are currently the only products that have been shown to improve human and animal health by combating pathogenic microorganisms and strengthening the immune system (Yeşilyurt et al., 2021).

Despite the availability of a wide range of useful microorganisms, the most common bacterial strains that are employed for the fabrication of probiotics are *lactic acid bacteria* (LAB), *Bifidobacteia, Propionibacteria*, *Saccharomyces boulardii* (yeast), and some Gram-negative bacteria like *E. coli* (Das et al., 2022). Of these, the experts from FAO and WHO advocated the use of *Lactobacilli* (lactic acid bacteria), and its numerous strains as probiotics (Zielińska et al., 2018). Table 2 provides an overview of probiotics’ effects on alcohol-induced liver damage in animal models. Most recent studies conducted on *Lactobacilli* and its species, and other microorganisms, for ALD therapy are as follows.

**TABLE 2:** A summary of Probiotic’s effects on alcohol-induced liver injury in Animal models.

Probiotic strain/formulation	Animal	Alcohol dosing	Duration of experiment	Effect with MOA	References
LAB					
*L. plantarum* microbeads (10^10^ CFU/mL)	Female Wistar rats (200–250 g	10 g/kg of body weight/day of 35% (v/v); oral gavage	12 weeks	↑↓ Endotoxins and hepatic marker enzymes	Rishi *et al.* (2017)
				↓ in various signaling molecules like COX-2, TLR4, MD2, CD14	
				↓ in TNF-α, IL-12 and NF-kB/p50	
L. plantarum CMU995 (1 × 10^10^, 6 × 10^9^, and 2 × 10^9^ CFU L)	male C57BL/6 J mice (22–25 g) w	1.25% (v/v) (initial conc.), 5% (v/v) alcohol (second week)	7 weeks	↓ in ALT, AST, TG, cholesterol	Fang *et al.* (2019)
				↓ endotoxins	
				↓ TNF-α, IL-1β	
				↑ Antioxidant parameters such as SOD, GSH, etc.	
*L. plantarum* ZS62 (1.0 × 109 CFU/Kg BW, p.o)	Male Kunming mice (Forty, 6-week-old)	56% alcoholic beverage, v/v) was given at 0.13 mL/10 g BW	8 days	↓ biomarker enzymes (ALT, AST)	Gan and Chen (2021)
				↓ hyaluronidase and precollagen III	
				↓ cytokines level	
				↑ antioxidant capacity	
*L. plantarum* HFY09 (1.0 × 109 CFU/Kg BW)	-	-	-	↓TG, TC, AST, ALT, precollagen III and hyaluronidase	Gan and Tong (2021)
				↑ ADH and ALDH activity	
				↑ SOD, CAT and GSH	
				↑ PPARα activity	
				↓ COX1, TLR-4, JNK and ERK expressions	
*L. plantarum*	Male Sprague-Dawley rats	-	4 weeks	↓ ALT, LPS, IL-6, and TNF-α, hepatic MDA levels	Chayanupatkul *et al.* (2022)
				↓TLR-4 expression	
*L. plantarum* C88 10^8^, 10^9^, and 10^10^ CFU/mL	Male ICR mice (18–22 g)	50% (v/v) ethanol (5 mL/kg/day/bw)	30 days	↓TNF-α, IL-6, and IFN-γ	Zhao *et al.* (2017)
				↓MDA levels	
				↑SOD activity by upregulation of Nrf2	
				Improved gut leakiness by overexpression of tight junctions	
				↓Endotoxin levels by downregulation of p38 and NF-kB	
*L. plantarum ZS62*	Mice	-	-	↓TNF-α, IL-6, and ↑IL-10 level	Wu *et al.* (2021)
				↑SOD, GSH, GSH-Px activity	
				↑ PGE2 and SS levels	
				Upregulation of Nrf2	
*L. rhamnosus* GG (2 × 10^10^ CFU/mL)	Male Wistar rats (225–250 g)	Initially 8 g/kg/day and increased up to 17 g/kg/day	2–2 and half weeks	↓ endotoxins	Nanji et al. (1994)
*L. rhamnosus* GG (2.5 × 10^7^ CFU/Kg BW)	Male Sprague-Dawley rats	(8 gm/kg) Twice daily	10 weeks	Improved gut permeability	Forsyth *et al.* (2009)
				↑ antioxidant parameters	
				↓ inflammatory markers	
*L. rhamnosus* GG (10^9^CFU/mouse per day)	C57BJ/6N mice	5% alcohol	8 weeks	Restored the levels of HIF-2α and intestinal trefoil factors	Wang *et al.* (2011)
LGG granules (0.05, 0.1, 1.0 g/(day-mouse)	Male C57BL/6N mice aged 8 weeks	35.5% as either alcohol	8 weeks	↓ LPS and TNF-α levels	Gu *et al.* (2020)
				↓ TG and MDA levels	
				↑ *Lactobacillus* and *bifidobacterium*	
LGG exosomes nanoparticles (2 × 10^9^ CFU/mL)	C57BL/6 J mice (6–8 weeks of age)	1.6%–5% alcohol (v/v) for 6 days and rest 5% for the subsequent 10 days	10 days	↑ tight junction proteins	Gu *et al.* (2021)
				↑ intestinal barrier function	
				Modulated AhR signaling	
				Upregulated Reg3 and Nrf2 expression	
*Lactobacillus rhamnosus* CCFM1107	Mouse model	-	-	↓ aminotransferase activity and endotoxins	Tian *et al.* (2015)
				↓ MDA	
				↓ in enterococci and enterobactor	
				↑ GSH, GSH-Px, and SOD	
				↑ lactobacilli and bifidobacterium	
*L. reuteri* (2 × 107 CFU, daily)	Male C57BL/6 mice (8–10 weeks of age)	(0.25 g/mL ethanol, 5 g/kg of body weight) weekly	8 weeks	↓ AST and ALT	Zheng *et al.* (2020)
				↓ TG and TCH	
				↑ IL-10	
				Downregulated NF-kB and MAPK signaling pathways	
*Lactobacillus fermentum* (10^9^ CFU)	Female mice (BALB/cAnNHsd) (12-month-old)	100 µL	12 weeks	↓ Hsp60, iNOS levels	Barone *et al.* (2016)
*L. fermentum* LA12	Rat model	-	4 weeks	↑ m RNA expression in TJ proteins	Kim *et al.* (2017)
				Improves intestinal barrier functions	
*L. brevis* (SBC8803) (1.45 × 109 CFU)	Male C57BL/6N mice (7-week-old)	-	5 weeks	↓ AST and ALT	Segawa *et al.* (2008)
				↓ TG and TCH	
				Downregulation of TNF-α, SREBP-1, and SREBP-2 mRNA	
*Bifidobacterium*					
*Bifidobacterium breve* ATCC_15700_ (10^10^ cells)	Eight-week-old adult male C57BL/6 mice (22 ± 2 g)	(3.8 g/kg body weight, 200 µL)	6 weeks	Improved immune homeostasis	Tian *et al.* (2020)
				Decreased endotoxemia	
				Stimulated intestinal barrier function	
				Overexpression of TJ proteins	
				Altered the gut microbiota	
*B. animalis* ssp. *Lactis* Probio strain; M8	Male Wistar rats (8-week-old)	Initially 1% ethanol, then increased 5% conc	6 weeks	↓ inflammation and oxidative stress	He *et al.* (2022)
				Restored the gut microbiota composition	
*Akkermansia muciniphila*					
*Akkermansia muciniphila* (1.5 × 10^9^ CFU) (Orally administered)	C57BL/6 female mice (7-8-week-old)	1–5 vol%	15 days	Reduced gut leakiness Enhanced mucus thickness and TJ expression	Grander *et al.* (2018)
*Roseburia spp*					
*R. intestinalis*	Mice	-	-	↑ IL-22 and REG3γ	Seo *et al.* (2020)
				↑ TJ protein	
*Lactococcus*					
*L. lactis NZ3900* (1 × 10^9^ CFU/mL)	Male mice (8-week-old)	56%v/v (dose = 5.6 g/kg body weight (BW)	15 days	↓ALT, AST and ALP	Lyu *et al.* (2018)
*L. chungangensis* CAU 1447 dry cells (1 × 10^9^CFU)	Male Sprague-Dawley rats (5-week-old)	50% alcohol at 4 g/kg BW daily	10 weeks	Reduction in ALP, AST, ALT and TG levels	Nam *et al.* (2019)

### 5.1 Lactic acid bacteria as probiotics

*Lactic acid bacteria* (LAB) are a heterogeneous group of bacteria that have been isolated from human (stomach, intestine, breast milk, and fecal matter), animal (pigs, calves, goats, fishes, and bees), fermented food (milk and dairy products, meat products, vegetables, cereals), non-fermented food (fruits and vegetables), and environment (food waste, soil, air) (Zielińska et al., 2018). LAB and its many species play a key role in food, agriculture, medical, and clinical fields (Harzallah and Belhadj, 2013). Many research publications have shown that LAB exhibit potent hepatoprotective action against alcohol, thioacetamide, CCl4, and tert-butyl hydroperoxide-induced hepatic damage in cultured cells and experimental animals (Han et al., 2005; Ou et al., 2012; Jantararussamee et al., 2021; Lee et al., 2021). Regarding to the mechanism of action, LAB maintains the balance of gut microorganisms, and improve gut permeability, resulting in a reduction in lipopolysaccharide levels and inflammatory responses. Moreover, oxidative stress is also reduced in the body (Sun et al., 2022).

A large body of evidence has corroborated specific strains of LAB, including *Lactobacillus* acidophilus, L. casei, L. rhamnosus, L. delbrueckii subsp. bulgaricus, L. brevis, L. johnsonii, L. plantarum*,* and L. fermentum*,* which are effectively used as probiotics against alcohol-induced liver injury by modulating the gut microbiome (Kechagia et al., 2013; Chen et al., 2022).

#### 5.1.1 Lactobacillus plantarum

A well-known strain offers health-promoting effects, particularly against liver disorders. A study was conducted involving the development and assessment of a microstructured synbox containing *L. plantarum* and epigallocatechin gallate (EGCG) for the treatment of alcohol-induced liver disease. A single delivery of formulated microbeads has shown a promising synergistic effect on endotoxins, alcohol, and hepatic enzymes. Furthermore, the synbox system diminished the levels of various signaling molecules such as COX-2, TLR4, CD14, MD2, and biomarkers such as TNF-α, IL12/p40, and NF-kB/p50 (Rishi et al., 2017). According to another study, supplementation of *L. plantarum* CMU995 significantly decreased the levels of aspartate aminotransferase (AST), alanine aminotransferase (ALT), triglycerides (TG), total cholesterol (TC), endotoxins, and inflammatory mediators (TNF-α, IL-1β) in an alcohol-induced liver injury model. Also, the concentration of antioxidant enzymes [superoxide dismutase (SOD), glutathione (GSH)] was found to be higher in *L. plantarum*-treated animals. The protective effect of this probiotic could be attributed to its ability to inhibit the migration of endotoxins into the blood and liver and further improvement in intestinal barrier function (Fang et al., 2019). Recently, the protective effect of *L. plantarum* ZS62 (LP-ZS62), a newly isolated strain from yoghurt, has been demonstrated using an alcohol-induced subacute liver injury model. In mice fed with the *Lactobacillus* strain (1.0 × 10^9^ CFU/Kg BW), reduction in biomarker enzymes, hyaluronidase, precollagen III and inflammatory cytokines and an increase in antioxidative status were observed. Notably, the protective effect of this strain could be due to its antioxidative and anti-inflammatory properties (Gan and Chen, 2021). *L. plantarum* HFY09 has also been shown to have protective effects in mice on ethanol-induced liver injury. The strain (1.0 × 10^9^ CFU/Kg BW) significantly decreased TG, TC, AST, ALT, precollagen III, and hyaluronidase and increased the levels of ADH and ALDH. Furthermore, antioxidant enzyme levels such as SOD and GSH were also upregulated, while downregulation of MDA was observed in mice fed with the probiotic strain. Notably, increased expression of peroxisome proliferator-activated-receptors α, glutathione peroxidase (GSH-Px), SOD 1 and 2, CAT, and NADPH and decreased expression of COX1, JNK, and ERK are the major mechanistic hallmarks for the protective effect of *L. plantarum* (Gan and Tong, 2021). More recently, the beneficial effects of *L. plantarum* against alcohol-induced liver injury were investigated using a rat model. The findings revealed a significant reduction in ALT, LPS, and inflammatory mediators (IL-6, TNF-α) in probiotic treated animals. Furthermore, downregulation of TLR-4 and MDA expression was also observed. Besides, fecal microbiota analysis indicated a significant enrichment of *Allobaculum*, and *Bifidobacterium*, however, *Romboutsia* and *Akkermansia* abundances were remained unchanged after probiotic treatment (Chayanupatkul et al., 2022). In chronic alcohol-induced liver injury in a mouse model, *L. plantarum* C88 caused substantial reductions in aminotransferases (ALT, AST), inflammatory markers (TNF-γ, IL-6, and IFN-γ), MDA levels, and an increase in the activity of antioxidant enzymes (SOD) in the liver. Downregulated hepatic CYP2E1and upregulated expression of nuclear factor erythroid 2-related factor 2 (Nrf2) were found to be key players in the protective effects of this probiotic strain. Moreover, improvement in gut leakiness *via* increased status of tight junction proteins, reduction in endotoxins-induced inflammation through downregulation of P38 phosphorylation and NF-kB were also the potential mechanisms implicated in the hepatoprotection of the *L. plantarum* C88 strain (Zhao et al., 2017). Another strain, namely, LP-ZS62, isolated from naturally fermented yak yoghurt, was found attenuated from alcohol-induced gastric injury. LP-ZS62 was found to suppress the content of MDA and enhance the activity of SOD and GSH in gastric tissues. Furthermore, GSH-Px, prostaglandin E2 (PGE2), and somatostatin (SS) levels were elevated by LP-ZS62. Besides, this probiotic abrogated the increased levels of cytokines IL-1α, TNF-α, and IL-6 and upregulated the level of the anti-inflammatory cytokine IL-10. Additionally, mRNA expression of Nrf2, copper/zinc SOD1, manganese SOD2, CAT, γ-glutamylcysteine synthetase (GSH1), and GSH-Px levels were enhanced by LP-ZS62, confirming that it protects ethanol-induced gastric injury through an antioxidant mechanism (Wu et al., 2021). Overall, *L. plantarum* has been proven to be a promising candidate for the treatment of alcohol associated hepatic disorders.

#### 5.1.2 Lactobacillus rhamnosus

Another species of *Lactobacillus*, namely, *L. rhamnosus*, has also been shown to halt the progression of ALD. As described earlier, endotoxins produced by many pathogens may trigger the development of ALD. A previous study has shown that *L. rhamnosus* GG (10^10^CFU) treatment significantly reduced the level of endotoxins and alleviated alcohol-induced hepatic injury in rats (Nanji et al., 1994). According to one study, supplementation with live *L. rhamnosus* (2.5 × 10^7^CFU) as probiotics significantly ameliorated alcohol-induced liver injury in a rat model of ASH. Besides, this bacterial strain improved gut permeability by reducing gut leakiness, oxidative stress, and inflammation in both the liver and intestine, thereby alleviating ASH in experimental rats (Forsyth et al., 2009). LGG was administered to C57BJ/6N mice for 2 weeks. Alcohol-induced endotoxemia and hepatic steatosis were mitigated after treatment, with a significant improvement in liver function. Moreover, alcohol-induced HIF and ITF levels were resorted, which are crucial elements for the development of ALD (Wang et al., 2011). In another study, LGG granules engineered by adopting the fluid bed granulation method recently demonstrated a remarkable protective effect against alcohol-induced liver damage. Serum lipopolysaccharide and TNF-α levels were suppressed. In addition, the granules also lessened TG, free fatty acids, and MDA production in the liver, which assisted to relieve hepatic steatosis. Subsequently, the proportion of beneficial bacteria like *lactobacillus* and *Bifidobacterium* was restored by LGG granules treatment (Gu et al., 2020). Another study demonstrated the protective effects of *L. rhamnosus* CCFM1107 against alcohol-induced liver injury in mouse model. The probiotic strain was shown to supress the levels of endotoxins, ALT, TG and cholesterol, while enhanced the levels of various antioxidant parameters like GSH, SOD and GSH-Px. The protective effect of *L. rhamnosus* CCFM1107 was found superior to that of LGG and to the drug Hu-Gan-Pian (Tian et al., 2015).

On the other hand, intestinal barrier function was found to be improved by exosome-like nanoparticles fabricated by LGG. In this study, tight junction proteins (TJ-proteins) expression in epithelial cells was upregulated *via* reducing the LPS-induced inflammatory response in macrophages. In addition, alcohol-induced intestinal barrier dysfunction and liver steatosis were ameliorated in animals orally fed with LGG-derived exosomes. It was noted that the protective effect of these nanoparticles was modulated through AhR signaling, which increases intestinal interleukin-22-Reg3 and Nrf2 expression, thus improving the barrier function (Gu et al., 2021). It has been reported that over secretion of bile acids (BA) into the intestine stimulates fat absorption as well as serves as signaling molecules to regulate biological functions mediated through several receptors like the FXR. FXR activation enhances the level of intestinal fibroblast growth factor (FGF), which further interacts with its receptor FGFR in the liver and suppresses both BA synthesis and lipogenesis in humans and animals. Most importantly, upregulated expression of microRNA (miR) 194 induced by alcohol led to decreased FXR expression, which results in increased hepatic BA synthesis and lipogenesis in alcohol-fed mice. Notably, alcohol-induced miR 194 expressions negatively influence taurine metabolism *via* taurine upregulated gene 1 (Tug1). It has been demonstrated in research that *LGG*-engineered exosome-like nanoparticle supplementation abrogated gut taurine level *via* restoring gut microbiome, and that was accompanied by a reduction of miR194 and activation of the FXR-FGF15 pathway. All these cascades finally resulted into decreased BA synthesis and lipogenesis in alcohol-induced liver injury in mice (Jiang, 2022). The aforementioned studies support the notion that *L. rhamnosus* might be a key player in alleviating alcohol-induced liver diseases.

#### 5.1.3 Lactobacillus reuteri

The next probiotic strain, *L. reuteri*, is also receiving great attention by researchers in the management of ALD. However, studies on its protective effects on liver diseases are rare. According to one study, *L. reuteri* significantly ameliorated liver injury by decreasing the levels of AST and ALT. Additionally, TG and TC levels were also reduced by probiotic treatment in the ALD model. The risk of polyunsaturated fatty acid metabolism disorder was found to be reversed, as confirmed by metabolomics analyses. Numerous staining techniques detected that alcohol-induced steatohepatitis was suppressed after probiotic treatment. The probiotic allayed the expression of inflammatory markers and stimulated the levels of anti-inflammatory mediators (IL-10) by downregulating NF-kB and mitogen-activated protein kinase signaling pathways (Liu et al., 2012; Hsu et al., 2017; Zheng et al., 2020). Besides, in the ALD model, *L. reuteri* has also been reported to show protective effects against galactosamine-induced liver injury and high-fat diet non-alcoholic fatty liver disease (NAFLD) mediated through gut dysbiosis and p-AKT/mTOR/LC-3II pathways (Jiang et al., 2021; Seif el-Din et al., 2021). This study might be used to figure out *L. reuteri’s* protective effects and underlying mechanism of probiotics in alcohol-induced liver damage.

#### 5.1.4 *Lactobacillus* acidophilus

*Lactobacillus acidophilus* is popularly known for the treatment of liver diseases. Probiotic treatment using *L. acidophilus* LA14 was performed against D-galactosamine-induced liver injury in rats. Intraperitoneal injection of *L. acidophilus* (3 × 10^9^ CFU) caused a significant reduction in hepatic enzymes, inflammatory cytokines and macrophage inflammatory proteins like MIP-1α, MIP-3α, and MCP-1 responsible for liver inflammation (Lv et al., 2021). To date, *L. acidophilus* has not been investigated against alcohol-induced liver injury; thus, in future, the aforesaid study might be used as a reference for the prevention of liver injury.

#### 5.1.5 *Lactobacillus* fermentum

*Lactobacillus fermentum*, a key strain of the *Lactobacillus* species, has wide applications in biomedical and food preservation fields (Naghmouchi et al., 2019). It has health-promoting properties when consumed. Despite having antimicrobial, antioxidant, and anti-inflammatory activities (Mikelsaar and Zilmer, 2009), it plays a significant role in preventing liver injury. Although, a limited investigation on the protective effects of *L. fermentum* has been explored in animal models. Animals treated with *L. fermentum* have shown a marked decrease in nitrated proteins like Hsp60, iNOS levels, and steatosis score, thus preventing ethanol-induced liver damage in mouse model (Barone et al., 2016). Similarly, oral dosing of *L. fermentum* LA12 reduced intestinal nitric oxide and hyperpermeability in an alcohol-induced liver damage rat model of ASH. Moreover, this probiotic upregulated the mRNA expression levels of TJ proteins and improved intestinal barrier function, prevented leakage of endotoxins in the blood and prevented hepatic steatosis in the experimental animals (Kim et al., 2017). According to a recent study, *L. fermentum* KP-3 was exploited to ferment ginseng (*Panax ginseng*), and the effect of fermented ginseng was further evaluated against alcohol-induced liver injury in C57BL/6N mice. The experimental results revealed a significant reduction in the levels of serum AST, ALT, LPS, TG, and TC in mice treated with fermented ginseng for 8 weeks. Additionally, fermented ginseng inhibited *de novo* lipogenesis *via* activation of the AMPK pathway and blocked P38 phosphorylation through the mitogen-activated protein kinase (MAPK) pathway, which resulted in a decrease in hepatic inflammation (You et al., 2020). Taken together, these findings validate the use of *L. fermentum* as a potential therapeutic intervention in the treatment of ALD.

#### 5.1.6 *Lactobacillus* casei

Another species, known as *L. casei*, has rarely been reported to exhibit protective effects against alcohol-induced hepatic damage in animal models. Nonetheless, *L. casei* was clinically investigated to assess its effect on lipid metabolism and intestinal microbiota in patients with ALD. In this double-blind, randomized controlled study, a total of 158 participants with ALD were divided into three groups: the low-dose, high-dose, and positive control groups. Patients receiving probiotic treatment had promising improvements in lipid metabolism and intestinal microflora when compared with the positive control group (Li X. et al., 2021). Some studies have shown the protective effect of *L. casei* Shirota using different models’ like galactosamine and fructose-induced liver injury in mouse models (Fang et al., 2018; Yan et al., 2022). These studies could be beneficial for further investigating its anti- ALD effects.

#### 5.1.7 Lactobacillus brevis

*L. brevis*, another member of LAB, is a Gram-positive, rod-shaped, and obligatory heterofermentative bacterium. The bacterium produces lactic acid, ethanol/acetic acid, and carbon dioxide. *L. brevis* has been reported to be isolated from fermented cabbage, silage, and other fermented food materials (Feyereisen et al., 2019). The bacterium has the ability to grow at temp. 30°C and a wide range of pH, i.e., 4–6 (Feyereisen et al., 2019). To date, different strains of *L. brevis* have been investigated for its antimicrobial, antioxidant, oral infection, and antagonistic activity against foodborne pathogens (Jang et al., 2019; Kariyawasam et al., 2020). Nevertheless, scant data is available on its potential therapeutic effect on alcohol-induced liver diseases in both animal and human models. The hepatoprotective effect of heat-killed *L. brevis* SBC8803 was assessed in ethanol-containing diet-fed C57BL/6N mice. *L. brevis* (100 and 500 mg/kg), once a day for 35 days, was administered in experimental animals, and several parameters were investigated. The finding revealed a substantial reduction in liver enzymes (ALT and AST), lipid profiles (TG and TC), and overexpression of TNF-α, sterol regulatory element-binding protein-1 (SREBP-1), and sterol regulatory element-binding protein-2 (SREBP-2) mRNA in the liver. Furthermore, heat shock proteins 25 (HSP25) mRNA expression in the gut was found to be upregulated as well (Segawa et al., 2008).

In another study, the efficacy of *L. brevis* SBC8803 was assessed on γ-glutamyl transferase in Japanese habitual drinkers. In this randomized, double-blind, placebo-controlled clinical study, subjects with high levels of gamma-glutamyl transferase (GGT) (50–100 IU/L) were treated with capsules containing live *L. brevis* SBC8803 for 8 weeks. The findings indicated a significant decrease in GGT (oxidative stress marker) and TG levels in the probiotic treated group as compared to the placebo group. This study supports that the probiotic supplementation may attenuate alcohol-induce oxidative stress and lipid metabolism (Wakita et al., 2012). However, more research on this probiotic strain is needed to explore its preventive effect against ALD along with its underlying mechanisms.

### 5.2 Bifidobacterium as probiotics

The genus *Bifidobacterium* is another representative member of commercially used bacteria that is believed to exert beneficial health promoting effects (O’Callaghan and van Sinderen, 2016). *Bifidobacterium* accounts for approximately 25% of the arable fecal bacteria in adults and 80% in infants (Picard et al., 2005). These are Gram-positive, rod-shaped, non-motile, non-spore forming, and pleomorphic anaerobic types of bacteria inhabiting both animal and human intestinal tract. Tissier (1899) first extracted one *Bifidobacterium* strain from the feces of newborn infants, which was designated as *Bacillus bifidus communis* (Carl and Patel, 2014). Subsequently, other strains have been isolated from various ecological niches, including sewage, oral cavity, the insect and mammalian gut, dairy products, and currently, water kefir. Recently, tremendous attention has been focused on this particular genus due to its health promoting benefits, and it is being incorporated as a main ingredient in numerous functional foods. Several studies validate its extensive use in the treatment of enteritis, constipation, brain disorders, infections, cancer, and many other conditions. Among the many *Bifidobacterium* strains, *B. infantis*, *B. longum*, and *B. bifidum* are widely exploited as probiotics (Chen Y. et al., 2021).

*Bifidobacterium* has been proven as a potential candidate against liver diseases, including both non-alcoholic and alcoholic diseases. An experimental study carried out in a group of children has demonstrated that various strains, *viz. B. longum*, *B. bifidum*, and *B. adolescentis* exhibit notable protection against NAFLD and obesity (Putignani et al., 2018). On the other hand, *B. longum* R0175 as probiotic offered a remarkable hepatic protection against D-galactosamine-induced acute liver failure in a rat model (Wang K. et al., 2020). Moreover, NAFLD induced by a high-fat and high-cholesterol diet in C57BL/6 J mice was prevented by *B. adolescentis* and *L. rhamnosus* mediated by various gut microbiota-dependent pathways (Wang G. et al., 2020). Another recent study conducted on a newly isolated strain, namely, *B. animalis* subsp. *lactis* V9, ameliorated NAFLD by modulating *de-novo* lipid synthesis and allaying inflammatory reactions *via* multiple signaling pathways, including the TLR-NF-kB and AMPK pathways (Yan et al., 2020). Other studies have demonstrated that two probiotic *Bifidobacterium*, namely, LI_09_ and LI_10_ alleviated liver injury by modifying vital members of the gut microbiota in rats (Zha et al., 2020). The aforementioned studies are associated with the effectiveness of *Bifidobacterium* strains against NAFLD.

So even though scientific evidence on the potential effects of *Bifidobacterium* on alcohol-induced liver diseases is limited, these studies may be useful in gathering vital insights on *Bifidobacterium’s* use in ALD.

Recently, the protective effect of *Bifidobacterium breve* ATCC_15700_ as a probiotic was evaluated against hepatic damage as well as gut microbiota in mice treated with high alcohol intake. Orally administered probiotics caused a significant reduction in endotoxemia, improved immune homeostasis, and stimulated intestinal barrier functions by accelerating the expression of TJ proteins in experimental animals. Furthermore, ATCC_15700_ recuperated the structure and composition of the gut microbiota (Tian et al., 2020). According to a more recent report, probiotic-fermented milk containing *B. animalis* ssp. Lactis Probio strain; M8 was assessed for its protective effect against alcoholic liver disease in rats. This study involved the determination of various biochemical parameters, such as the estimation of proinflammatory mediators, liver function-related indicators, and antioxidant indicators. The results illustrated that animals fed with probiotics substantially allayed liver inflammation, oxidative stress and improved the gut microbiota’s stability as well as alleviated hepatic injury in ALD. Furthermore, alcohol-induced dysbiosis was ameliorated by restoring the gut microbiome composition. Probiotic intervention also increased the levels of fecal metabolites such as tryptophan, cortisol, vitamin K2, and inositol, as noted by fecal metagenome study (He et al., 2022).

It is clear from various experimental studies that *Bifidobacterium* strains could attenuate both NAFLD and alcohol-induced liver damage. It is worth noting that additional research on liver injury caused by prolonged alcohol consumption is needed on a variety of distinct strains.

### 5.3 *Saccharomyces boulardii* as probiotics

*Sacchromyces boulardi*, a non-pathogenic yeast, is gaining popularity as probiotics for a variety of disorders and exerting significant benefits to human health (Tiago et al., 2012). *S. cerevisiae* (Baker’s yeast), on the other hand, is not anticipated to have the same lucrative effects on humans as *S. boulardi* (Tiago et al., 2012). Henri Boulard, a French scientist, was the first to isolate *S. boulardi* from lychee and mangosteen fruits in 1923. Due to the presence of unique and distinctive traits such as thermostability, acid tolerance, pH resistance, bile salt tolerance, and resistance to exposure to the stomach environment, this yeast is far superior to non-probiotic *S. cerevisiae* (Prajapati and Patel, 2013). Many literatures demonstrated that *S. boulardi* has been clinically and experimentally proven for the treatment of numerous acute (*H. pylori* and *Clostridium* infections, and diarrhea) and chronic diseases (Crohn’s disease. ulcerative colitis, and IBS) (McFarland, 2010; Kelesidis and Pothoulakis, 2012; Szajewska and Kołodziej, 2015; Kaźmierczak-Siedlecka et al., 2020). A recent study also claimed that *S. boulardi* as a biotherapeutic agent is effectively used to alleviate hepatic damage, hepatic steatosis, and liver fibrosis, as well as improve the liver functions (Everard et al., 2014; Li et al., 2014; Wu et al., 2014; Yu et al., 2017). This probiotic yeast is thought to work through a variety of mechanisms, including regulation of intestinal microbial homeostasis, interference with pathogens’ ability to colonize and infect the mucosa, modulation of local and systemic immune responses, stabilization of the gastrointestinal barrier function, and induction of enzymatic activity that promotes absorption and nutrition (Kelesidis and Pothoulakis, 2012).

It has been reported that an imbalance in the gut microbiome is one of the key factors associated with liver ailments. Hepatoprotective effect of *S. boulardi* was evaluated in D-galactosamine induced liver damage in a BALB/c mice model. Treatment with *S. boulardi* (1 × 10^9^ CFU/mL) caused a substantial decrease in ALT and AST levels in experimental animals. Histopathological investigations were also conducted and have shown normal architecture of the liver. The principle underlying mechanism implicated in hepatoprotection was notably alteration in the gut microbiome composition, predominantly by enhancing the bacterial content belonging to the families Bacteroidaceae and Clostridiaceae and lowering the proportion of bacteria of families Anaeroplasmataceae, Alcaligenaceae, Caulobacteraceae, and Rikenellaceae (Yu et al., 2017). In sum and substance, *S. boulardi* could be effectively used as a therapeutic candidate to mitigate the liver ailments*. Nevertheless, no research on this nonpathogenic yeast against alcoholic and non-alcoholic liver illnesses has been published yet, therefore, more research is required to fill this gap.*

### 5.4 Akkermansia muciniphila as probiotic

*Akkermansia muciniphila*, which resides as an intestinal symbiont in the mucosal layer, represents the next-generation of prominent probiotic species (Zhai et al., 2019). The bacterium is therapeutically employed in the treatment of diabetes, metabolic disorders, atherosclerosis, autism spectrum disorders, cancer, and immune-mediated diseases (Zou and Chen, 2020; Aron et al., 2021; Yan et al., 2021)**.** In 2004, Derrien and his workers first isolated this non-motile, oval-shaped, anaerobic Gram-negative bacterium from the fecal sample comprising gastric mucin as an energy source (Derrien et al., 2004; Zhang et al., 2019).

*A. muciniphila* is abundantly found in mammalian guts, accounting for 3%–5% of the microbial flora in the human gut. The bacterium exhibits a unique property to degrade mucin and competitively inhibits the growth of pathogenic microorganisms that degrade the mucin (Derrien et al., 2017; Chia et al., 2018; Hasani et al., 2021). *A. muciniphila* has received a lot of interest in the research world in recent years because of its potent probiotic capabilities, particularly against obesity and diabetes. (Zou and Chen, 2020; Hasani et al., 2021). Although little is known about the mechanistic potentials of *A. muciniphila* in alleviating several diseases. Nevertheless, recent papers have shed light on molecular mechanisms associated with its anti-inflammatory, neurological, and metabolic disorders (Si et al., 2022).

Despite being a promising probiotic candidate, *A. muciniphila’s* formulated preparations are not yet available across the globe. In addition, limited animal experiments and clinical studies have been conducted on this bacterium. In terms of mechanism, one study illustrated the ability of *A. muciniphila* to protect the intestinal mucosa from injury in chicks caused by *S. pullorum*. This could be possible through the Wnt/β-catenin signaling pathway, which initiates enhanced proliferation of intestinal cells and thus protects the intestinal barrier (Zhu et al., 2020). In high fat diet (HFD) and CCl4 induced liver injury in C57BL/6 mice, pasteurized *A. muciniphila* and its extracellular vesicles (EVs) supplementation ameliorated intestinal permeability, reduced inflammatory responses, and restored the fecal targeted bacteria composition, thereby alleviating the symptoms of liver fibrosis (Keshavarz Azizi Raftar et al., 2021).

Excessive alcohol consumption causes depletion of the microbial population of the gut, including *A. muciniphila*, which is beneficial for a healthy gut. A recent study was designed to demonstrate the effect of *A. muciniphila* on acute and chronic ALD in experimental mice and humans. In this study, oral administration with *A. muciniphila* significantly ameliorated ethanol induced hepatic injury by reducing gut leakiness, enhancing mucus thickness, and promoting TJ-proteins expression, thus preserving the intestinal barrier’s integrity (Grander et al., 2018). In a nutshell, *A. muciniphila* has been shown to have a protective effect against ALD in both animals and humans, suggesting that it could be employed as a promising probiotic for liver illnesses. Nevertheless, limited assessment of this bacterium may further pique the interest of researchers in the management of hepatic diseases.

### 5.5 *Escherichia coli* nissle as probiotics

*Escherichia coli* Nissle (EcN), a newly discovered strain of *E. coli*, was originally isolated in 1917 by Alfred Nissle from the feces of a soldier with no sign of infectious diarrhea. EcN is a non-pathogenic Gram-negative microorganism that confers a wide range of health benefits on human beings (Sonnenborn and Schulze, 2009; Scaldaferri et al., 2016; Sonnenborn, 2016).

Mutaflor, a branded preparation, is formulated using this active strain and is largely distributed in various countries like Australia, Europe, and Canada (Soundararajan et al., 2019; Pradhan and Weiss, 2020). Besides, Symbioflor 2 and Colinfant are some other commercially available products of *E. coli* strains (Jacobi and Malfertheiner, 2011; Naresh Kumar and Archana, 2021). EcN is effectively used in the treatment of neuroinflammatory disorders, gastrointestinal disorders like ulcerative colitis, diarrhea, and irritable bowel syndrome (Secher et al., 2017; Manzhalii et al., 2022; Zhao et al., 2022). A wide array of studies has corroborated that the therapeutic effects of this probiotic strain could be achieved in many ways, including modulating the immune system, ameliorating gut barrier function, and competing with pathogenic microorganisms for adhesion to mucosa (Plaza-Diaz et al., 2019). There is still a gap in establishing the mechanistic insights of *E. coli* as a probiotic.

One study demonstrated the *in-vitro* and *in-vivo* protective effect of fabricated EcN in the form of matrices consisting of curli nanofibers against dextran sodium sulfate-induced colitis in mice model (Naresh Kumar and Archana, 2021). As mentioned above, EcN is extensively researched for the treatment of intestinal diseases, but its mechanistic approaches to communicating with the host were not established. Therefore, a recent study on EcN-derived outer membrane vesicles (EcN-OMVs) was conducted to examine the immunomodulatory and antimicrobial effects in RAW 264.7 macrophages. This study clearly indicated that EcN-OMVs induced proliferation, immune-related enzymatic activities, and phagocytic functions in RAW264.7 cells. Moreover, EcN-OMVs induced more anti-inflammatory responses (IL-10) than pro-inflammatory responses (IL-6 and TNF-α) *in vitro*, and modulated the production of Th1-polarizing cytokines (IL-12) and Th2-polarizing cytokines (IL-4). Treatments with EcN-OMVs effectively improved the antibacterial activity of RAW 264.7 macrophages (Hu et al., 2020). In addition to the studies listed above, EcN has also been studied as a probiotic for the treatment of hepatic disorders (Hu et al., 2020). EcN (pqq-glf-mtlK) and EcN (pqq-fdh), genetically modified EcN, have recently demonstrated significant recovery of hepatic enzymes (AST, ALP, and ALT) as well as a diminution in lipid peroxidation and antioxidant enzyme activity in fructose-induced hepatic damage in rats (*Secher* et al.*, 2017*).

*Except for a few instances, the preventive efficacy of EcN against alcohol-induced liver injury has not been extensively studied. In a double-blind, randomized study conducted on 39 patients with liver cirrhosis, EcN treatment caused significant improvement in reducing the level of endotoxin, normalizing the intestinal colonization, and strengthening the liver functions* (*Lata* et al.*, 2007*)*. A more recent study has corroborated the protective effect of genetically modified* EcN 1917 *on alcohol–induced acute liver injury. In this context, EcN was first genetically engineered to express a number of genes, including ADH, ALDH, NAD synthase, and NADH oxidase.* Modified EcN treated mice have shown a reduction in elevated levels of hepatic marker enzymes (ALT, AST), MDA, TG, TNF-α, and IL-1β along with an increase in GSH and SOD levels, which could be attributed to reduced oxidative stress, lipid peroxidation and inflammation. Moreover, recovery of gut microbiota homeostasis was also noticed with EcN supplementation, which is responsible for detoxification of toxic alcohol metabolites in liver (Cao et al., 2021). Another study was carried out on a newly identified strain, namely, *E. coli.* Nissle-metallothionein (EcN-MT), attenuated cadmium-induced liver injury in mice. Moreover, the protective effect was mediated through upregulated levels of antioxidant enzymes and downregulated expression of TLR4, NF-kB, and myeloid differentiation factor 88 (Myd88) (Zou et al., 2022). The previously mentioned *E. coli* study may pave the way for scientific proof against alcohol-induced liver diseases.

### 5.6 *Roseburia spp.* as probiotic

Among many superstars as probiotics, the genus *Roseburia* also exerts probiotic activities that boost the heath of body. It was named after Theodor Rosebury, an American microbiologist who made significant contributions to the field of the oral microbiome (Stackebrandt, 2014). *Roseburia* spp. is popularly known to exhibit immunomodulatory effects, improve the gut microbiome ecology, and combat numerous human ailments (Tamanai-Shacoori et al., 2017). *Roseburia* spp. comprises about 7%–24% of the total bacteria in the human colon. Interestingly, like other bacteria, *Roseburia* is able to transform monosaccharides into micro metabolites in the form of short chain fatty acids like acetate, propionate, and butyrate. Out of these three, butyrate is considered as a key source of energy for the human colon. Additionally, this metabolite is known for its anti-cancer, anti-inflammatory, and gut protective properties in the distal gut (La Rosa et al., 2019). Notably, a reduced abundance of butyrate-producing bacteria triggers inflammatory disorders and increases the risk of colon cancer (Geng et al., 2013; Takahashi et al., 2016).

*Roseburia intestinalis*, Roseburia *hominis*, *Roseburia inulinivorans*, Roseburia *faecis*, and *Roseburia cecicola* are all well-known species that produce short chain fatty acids (Tamanai-Shacoori et al., 2017). Of these, *R. intestinalis* has achieved a considerable amount of attention in the research domain. Evidence points out that this bacterium is highly efficacious in the treatment of GIT disorders (IBD, ulcerative colitis), diabetes mellitus, atherosclerosis, neurological diseases, cardio and antiphospholipid syndrome (Nie et al., 2021). Complementary reports highlighted that colonization of the mucin layer by *R. intestinalis* enhances the availability of butyrate, which is beneficial for colonic epithelial cells (van den Abbeele et al., 2011). The underlying mechanisms for its therapeutic potential are still undefined. Nonetheless, anti-inflammatory action could be attributed to its ability to downregulate the expression of IL-7 and subsequently stimulate regulatory T cells (Treg) to prevent the risk of colitis (Zhu et al., 2018).

It is important to note that after prolonged use of alcohol, intestinal levels of short chain fatty acids are reduced, which further leads to an increase in toxic ethanol metabolites. In this context, consumption of the short chain fatty acids (SCFA) (butyrate) ameliorates gut barrier function (Hartmann et al., 2015).

According to a recent study, butyrate producing bacteria, i.e., *Roseburia* spp., halted the progression of alcoholic fatty liver in the ALD murine model. The data revealed that the bacterium ameliorates both hepatic steatosis and inflammation and maintains the gut microbiota balance. Moreover, restoration of the gut microbiome by *R. intestinalis* could be profoundly attributed to upregulation of IL-22 and regenerating islet-derived protein 3 gamma (REG3γ). In addition, modulation of TLR5 recognition and elevated status of the TJ protein (Occludin) are the other mechanisms through which *R. intestinalis* improves the gut ecosystem (Seo et al., 2020). In summary, *Roseburia* could be a potential candidate for the treatment of ALD, but more research is warranted to explore its effect on alcohol-induced liver injury along with associated molecular mechanisms.

### 5.7 *Lactococcus* as probiotic

A variety of *Lactococcus* members (belonging to the LAB family) have been proposed and used as probiotic strains in order to boost human health. These are abundantly exploited as starter bacteria specifically in the manufacturing of fermented products, such as cheese and yogurt (Jung et al., 2020). In contrast to other bacteria including LAB, *Bifidobacterium*, etc., limited investigations on the probiotic effects of *Lactococcus* strains have been conducted since these are not considered to be natural inhabitants of the human GIT (Kimoto et al., 1999). The *Lactococcus* genus is a non-motile, non-sporulating, Gram-negative, and cocci-shaped bacteria (Jung et al., 2020). To date, two species within the genus, *Lactococcus lactis* and *Lactococcus chungangensis*, have been reported for their protective effects against alcohol-induced liver injury. *L. lactis* NZ3900 with ADH and ALDH activity has been reported to ameliorate acute alcoholic liver injury in mice. In this study, several serum hepatic biomarkers such as aminotransferases (ALT, AST, and ALP) were reduced by *L. lactis* recombinant at a high dose (ADH activity, 2000U/Kg; ALDH activity, 1000 U/Kg). Additionally, a reduction in ethanol-induced elevated lipid levels and oxidative stress was also observed (Lyu et al., 2018). Recently, another *Lactococcus* strain, namely, *L. chungangensis*, appeared to exhibit a protective effect against chronic alcoholic liver disease. Oral supplementation of *L. chungangensis* CAU 1447 dry cells and CAU 1447 cream cheese to rats caused a significant reduction in ALP, AST, ALT, and TG levels. Furthermore, probiotic treatment raised the level of short chain fatty acids, butyrate, and acetate in feces. The protective effect of this probiotic was mediated through anti-inflammatory and antioxidative mechanisms (Nam et al., 2019). Nevertheless, little is known in context to both *Lactococcus* strains, hence, more studies are required to explore them for the treatment of ALD.

### 5.8 Probiotic mixtures

A number of studies have proven that probiotic mixtures are more effective than a single strain in the treatment of a wide range of disorders (Chapman et al., 2011; Fong et al., 2022; Tracey et al., 2023). A *Lactobacillus* mixture containing *L. plantarum* KLDS1.0344 and *L. acidophilus* KLDS1.0901 was investigated for its protective effect using a chronic alcoholic liver lesion model. In this study, C57BL/6 J mice were provided with the Lieber-DeCarli liquid diet containing alcohol for 6 weeks. Notably, oxidative stress, inflammation, and lipid accumulation were significantly reduced, possibly *via* AMPK, Nrf-2, and TLR4/NF-kB pathways. Furthermore, the *Lactobacillus* mixture altered the gut microbiota composition and decreased the number of pathogenic microorganisms. An increase in the levels of short-chain fatty acids and a decrease in the serum lipopolysaccharide levels were also observed, which further contributed to improving the intestinal permeability (Li H. et al., 2021). More recently, many *Lactobacillus strains*, including *Levilactobacillus brevis* (MG5280 and MG5311), *Limosilactobacillus reuteri* (MG5458), and *Limosilactobacillus fermentum* (MG4237 and MG4294), were evaluated for the protective effect against alcohol-induced HepG2 cells. Amongst various strains, only five strains, namely, *L. brevis* (MG5280 and MG5311), *L. reuteri* (MG5458), and *L. fermentum* (MG4237 and MG4294), have shown a protective effect against liver injury, which could be attributed to the regulation of CYP2E1, lipid synthesis factors (SREBP1C and FAS), lipid oxidation factors (PPAR*α*, ACO, and CTP-*1*), and antioxidant enzymes (CAT, SOD, and GPX). Furthermore, these probiotic strains were found safe, as confirmed by antibiotic susceptibility and hemolysis assays (Lee et al., 2021). Subsequently, a positive therapeutic effect of *L. plantarum* along with other *Lactobacillus* sp. (*L. fermentum* and *L. reuteri*) was also noticed against ASH and liver damage. The levels of AST, ALT, TG, and other proinflammatory markers like TNF-α, IL-6 were significantly downregulated in mice fed with three probiotic strains. In addition, upregulation of GSH and GSH-Px activity was also observed, which are the key biomolecules of oxidative stress in the liver (Hsieh et al., 2021).

*Lactobacillus* species have also been used as probiotics in combination with other bacterial strains. For instance, in alcohol-induced ALD in a mouse model, the probiotics containing *L. rhamnosus* GKLC1, *L. casei* GKC1, *L. plantarum* GKM3, *L. paracasei* GKS6, and *Bifidobacterium lactis* GKK2 were orally fed at a dose of 0.82 g/kg B.W. for 8 weeks. The probiotic significantly alleviated ALD by reducing the levels of serum enzyme (ALT) and lipid profile (TG, TC) (Tsai et al., 2020). The biological effects of probiotic mixtures (*Lactobacillus rhamnosus* R0011 and *acidophilus* R0052), KRG (Korea red ginseng), and urushiol (*Rhus verniciflua* Stokes) on ALD, including their effects on a normal and high-fat diet in C57BL/6 mice, were extensively investigated. The results demonstrated that probiotic mixtures, KRG, and urushiol significantly reduced the levels of TNF-α, and IL-1β. Besides, alcohol-induced TLR 4 expression was downregulated by probiotics in the normal and high-fat diet groups. Interestingly, another study has demonstrated the effect of multi-species probiotic supplementation on alcohol and acetaldehyde metabolism in rats. High tolerance for both alcohol and acetaldehyde were observed in only four probiotic species, namely, *L. casei* CBT LC5, *L. gasseri* CBT LGA1, *Bifidobacterium lactis* CBT BL3, and *Bifidobacterium breve* CBT BR3, which can also be regarded as ProAP4. In addition, these species also exhibited high mRNA expression of alcohol and ALDH. Moreover, rats fed with ProAP4 (probiotics and excipients) for 2 weeks showed reduced concentrations of alcohol and aldehyde in the serum. Also, aminotransferase activity was found to be decreased, suggesting that these four probiotic strains exert protective effect against alcohol-induced liver injury (Lim et al., 2021).

These compositional studies restored hepatic health and warrant the need for future investigations to root out their hepatoprotective potential against ALD.

## 6 Clinical evidences of probiotics

Today’s scenario represents a sudden upsurge in widespread acute to chronic disorders, meanwhile, exhaustive and innovative research is becoming more significant in order to fight against such multifaceted diseases. Despite the tremendous discovery of conventional medicines, probiotics are now captivating the attention of researchers as they have been experimentally proven for the treatment of an enormous range of human illnesses, including GIT disorders, cardiovascular disorders, neurological disorders, and many more. Undoubtedly, probiotics and related products, by altering the gut ecosystem, may protect against enteric problems, and maintain the overall health (Markowiak and Ślizewska, 2017). It is noteworthy that probiotics, due to their heterogenic property, differ in their composition, dose, and therapeutic effects among various formulated products (Michail et al., 2006). Therefore, clinical studies are necessary in order to determine the safety and efficacy of probiotics or probiotic preparations. As per recent literature, more than 1000 clinical trials with probiotics have been conducted for over 700 different disease conditions and are registered at ClinicalTrials.gov and/or the International Clinical Trials Registry Platform (ICTRP) of the WHO. Among various probiotic strains, LGG and *Bifidobacterium animalis* spp. are the most widely clinically tested strains (Dronkers et al., 2020; Dudek-Wicher et al., 2020).

In the context of ALD, many reports have shown the promising effects of probiotics using experimental animal models, as discussed in previous sections. This segment proceeds with some clinical investigations of probiotics, however, these are limited. Table 3 summarizes various clinical evidences conducted on probiotics against ALD (Li et al., 2016).

**TABLE 3:** Clinical evidences on probiotics.

Aim	Design	Probiotic treatment/duration	Participants	Outcomes	References
Effect on liver damage in patients with various types of liver diseases along with estimation of cytokines and oxidative stress parameters	Open study	VSL#3/	Total: Alcoholic cirrhosis patients = 20 NAFLD patients = 22 HCV-positive = 36	Improvement in plasma levels of MDA, 4-hydroxynonenal (4-HNE) in both NAFLD and AC patients	Loguercio *et al.* (2005)
				Reduction in cytokines levels (TNF-α, IL-6 and IL-10) in AC patients only	
				No effect on HCV patients	
				Improvement in S-nitrosothioles (S-NO) in all groups	
Effect on the intestinal flora, endotoxin level and liver functions in liver cirrhosis patients	Double-blind, randomized	*E. coli.* Nissle (Mutaflor)/42 days	Liver cirrhosis patients = 39	Restored normal microbiome in the gut	Lata *et al.* (2007)
				Reduced endotoxin levels	
				Ameliorated liver functions as evaluated by Child-Pugh score	
Evaluation of efficacy in preventing the recurrence of hepatic encephalopathy [HE] in cirrhosis patients	Double-blind, randomized	VSL#3 9 × 10 (11) bacteria/6 months	Total = 66 Placebo = 64	Alleviated HE in treated group (34.8%) and placebo (51.6%)	Dhiman *et al.* (2014)
				Improved Child-Turcotte-Pugh (CTP) and model for end-stage liver disease (MELD) scores	
Effect on deranged neutrophil function and cytokine response in alcohol cirrhosis patient	Open-label study	*Lactobacillus casei* Shirota (6.5 × 109); three times daily/4 weeks	AC patients = 12 Healthy controls = 13	Recovery of neutrophil phagocytic capacity	Stadlbauer *et al.* (2008)
				Lowering of sTNFR1, sTNFR2 and IL10 levels	
				Upregulation of TLR2, 4 and 9	
Probiotic effect on liver damage against various types of liver diseases		LAB (2 cps/die)/2 months	AC patients = 10	Reduced ALT, AST and GGT levels	Loguercio *et al.* (2002)
				Improved TNF, IL-6, and IL-10 levels	
				Supressed MDA, 4-HNE and S-NO levels	
Effect of probiotics on the intestinal flora against alcohol-induced liver injury	A randomized-pilot study	*Lactobacillus plantarum* 8PA3 and *Bifidobacterium bifidum*/5 days	Total patients = 66	Reduction in AST, ALT, LDH, GGT and total bilirubin	Kirpich *et al.* (2008)
				Improvement in the gut microbial flora	
Probiotic effect on liver function in alcoholic cirrhosis patient	Double-blind placebo-controlled	Yakult 400 (*L. casei* Shirota; twice a day)/4 weeks	Total ALC patients = 37	Increase in serum transthyretin levels in Y400 treated group	Koga *et al.* (2013)
			Y400 (n = 18)	Decrease in hypersensitive C-reactive protein	
			Placebo (n = 19)	Intestinal microbiota balance	
				Improvement in liver functions	
Assessment of probiotic efficacy in allaying the gut bacterial overgrowth and permeability in chronic liver disease	Randomized study	Lactowel/	Toatl patients = 53	Increase in B. lactis, L. rhamnosus, and L.acidophilus content in the feces	Kwak et al., 2014; Hong et al., 2019; Vatsalya (2021)
				Alleviate bacterial overgrowth	
				Improvement in gut intestinal pearmeability, but less than placebo	
				Improvement in overall GI symptoms	

## 7 Conclusion

Conclusively, this review has provided a comprehensive outlook on innovative therapeutic strategies for alcohol-induced liver diseases. Many literatures have demonstrated that overconsumption of alcohol and its toxic metabolites trigger the development of broad-spectrum liver disorders. Among several causative factors for ALD, gut microbiota imbalance has been shown to be a significant factor in the progression of disease. It has been reported that alcohol and or/acetaldehyde profoundly altered the composition of the gut ecosystem, which may further contribute to disrupting the intestinal barrier function and increasing the permeability. Meanwhile, increased gut permeability led to the gut leakiness, translocation of pathogenic bacteria and endotoxins, which could reach the liver *via* the portal vein system. Due to the absence of approved drugs, therapeutic interventions in the form of probiotics have received unprecedented attention to halt the progression of ALD. A plethora of probiotics constituting many bacterial strains and related products have been recognized for the treatment of ALD. A wide range of *Lactobacillus* strains have been widely investigated, eliciting great therapeutic potential for the prevention and treatment of ALD; while *Bifidobacterium*, *E. coli Nisseli*, *A. muciniphila*, and others are in the pipeline to gather scientific evidence against ALD. Despite multiple mechanisms such as reducing oxidative stress and the inflammatory response as well as improving intestinal barrier function, modulation and restoration of the intestinal microbiome is the most common mechanistic route by which commercial probiotics exert their protective effect against alcohol-induced liver disorders. Moreover, some probiotics have been clinically investigated to ensure their efficacy and safety profiles as well. Collectively, traditionally used probiotics and their products might be novel therapeutic interventions for a variety of ALD.

## 8 Future perspectives/interpretations

In the last few decades, there has been incessant research in the gut microbiome and its impact on human health and associated disorders. It is noteworthy that alcohol-induced alteration of the gut microbiota is one of the major hallmarks of ALD pathogenesis. These days, probiotics are being exploited as potential therapeutic interventions for the treatment of ALD. Probiotics directly or indirectly regulate the gut microbial composition, improve the intestinal barrier function, and thus alleviate liver injury, as claimed by various preclinical and clinical data. In addition, probiotics stimulate the production of microbial metabolites, including AhR, short-chain fatty acids, etc. which also contribute to modulating the gut microbiota composition. Subsequently, prebiotics, symbiotics and fecal microbiota transplantation (FMT) are some of the novel therapeutic approaches for ALD treatment. Despite a huge surge in research on the microbiome, its related liver disorders, and microbiome-based treatments, there is still a gap and/or limitations in this domain. Some of the following points related to future investigations have been illustrated below.

1. A variety of pathophysiological mechanisms related to ALD have been described in the previous section. However, the establishment of other routes connecting the gut microbiota to alcohol-induced liver disease is yet to be warranted. This may support a better understanding of the gut-liver axis and the fabrication of several therapeutic strategies/indications.
2. Although, many bacterial strains have been experimentally investigated (both animals and humans) for their potential hepatoprotective effects, It is worth noting that some other effective supplements, as probiotics, prebiotics, and FMT, and their formulations need to be researched. Furthermore, their pharmacokinetic/pharmacodynamic parameters are also worth detecting in the future.
3. Despite several associated mechanisms, much more attention is needed to explore the underlying mechanisms and other targets for probiotics to prevent ALD.
4. To date, a limited number of clinical trials have been conducted on specific probiotic strains using a small number of enrolled subjects. Also, probiotics safety and efficacious profile are inappropriate and uncertain. Therefore, a large number of clinical trials are warranted at large scale to determine the therapeutic effectiveness.
5. More insights into the formulation methods, fixing doses, and their uses alone or in combinations should also be considerable.

## Figures and Tables

**Table:** 

**ADH**	alcohol dehydrogenase
**AEAT**	acyl-CoA-ethanol-O-acyltransferase
**AhR**	Aryl hydrocarbon receptor
**AKT**	α-serine/threonine-protein kinase
**ALDH**	acetaldehyde dehydrogenase
**ALD**	alcohol-associated liver diseases
**ALT**	alanine aminotransferase
**AH**	alcoholic hepatitis
**AMPK**	AMP-activated protein kinase
**ASH**	alcoholic steatohepatitis
**ASK1**	apoptosis signal-regulating kinase 1
**AST**	aspartate aminotransferase
**AUD**	alcohol use disorder
**BA**	bile acids
**BAC**	Blood alcohol concentration
**CAT**	catalase
**CYP2E1**	cytochrome P450 2E1
**CPS**	capsular polysaccharides
**EcN**	*Escherichia coli* Nissle
**EGCG**	epigallocatechin gallate
**EGFR**	epidermal growth factor receptor
**EtG**	ethyl glucuronide
**EtS**	ethyl sulfate
**EVs**	extracellular vesicles
**FAEEs**	fatty acid ethyl esters
**FAO**	the United Nations food and agriculture organization
**FGF**	fibroblast growth factor
**FXR**	farnesoid X receptor
**GIT**	gastrointestinal tract
**GLUT 4**	glucose transporter-4
**GSK3-β**	glycogen synthase kinase 3 beta
**GSH**	glutathione
**GSH1**	γ-glutamylcysteine synthetase
**GSH-Px**	glutathione peroxidase
**HCC**	hepatocellular carcinoma
**HFD**	high fat diet
**HIF**	hypoxia-inducible factor
**HNE**	4-hydroxy-2-nonenal
**HSC**	hepatic stellate cells
**HSP25**	heat shock proteins 25
**IBS**	inflammatory bowel disease
**ICTRP**	international clinical trials registry platform
**iNOS**	inducible nitric oxide synthase
**ITF**	intestinal trefoil factor
**JNK**	c-Jun N-terminal kinases
**LAB**	lactic acid bacteria
**LGG**	*Lactobacillus* rhamnosus GG
**LP-ZS62**	*Lactobacillus* plantarum ZS62
**LPS**	lipopolysaccharides
**MAPK**	mitogen-activated protein kinase
**MDA**	malondialdehyde
**miR122a**	micro RNA 122a
**Myd88**	myeloid differentiation factor 88
**NAD+**	nicotinamide adenine dinucleotide
**NAFLD**	non-alcoholic fatty liver disease
**NF-kB**	nuclear factor-kappa B
**Nrf2**	nuclear factor erythroid 2-related factor 2
**PA**	phosphatidic acid
**PEth**	phosphatidylethanols
**PGE2**	prostaglandin E2
**PLD**	phospholipase D
**PRRs**	specific pattern recognition receptors
**REG3γ**	regenerating islet-derived protein 3 gamma
**ROS**	reactive oxygen species
**SCFA**	short chain fatty acids
**SLPs**	surface layer proteins
**SREBP**	sterol regulatory element-binding protein
**SOD**	superoxide dismutase
**SS**	somatostatin
**TC**	total cholesterol
**TfR1**	transferrin receptor 1
**TNF**	tumor necrosis factor
**TLR**	toll-like receptors
**Tug1**	taurine upregulated gene 1
**UDP**	uridine diphosphate
**UGT**	uridine diphosphate‐glucuronosyltransferases
**WHO**	world health organization
**XIAP**	X-linked inhibitor of apoptosis
